# Atomic and structural modifications of two-dimensional transition metal dichalcogenides for various advanced applications

**DOI:** 10.1039/d2sc01398c

**Published:** 2022-05-18

**Authors:** Balakrishnan Kirubasankar, Yo Seob Won, Laud Anim Adofo, Soo Ho Choi, Soo Min Kim, Ki Kang Kim

**Affiliations:** Department of Energy Science, Sungkyunkwan University Suwon 16419 South Korea kikangkim@skku.edu; Department of Chemistry, Sookmyung Women's University Seoul 14072 South Korea soominkim@sookmyung.ac.kr; Center for Integrated Nanostructure Physics (CINAP), Institute for Basic Science (IBS), Sungkyunkwan University Suwon 16419 South Korea

## Abstract

Two-dimensional (2D) transition metal dichalcogenides (TMDs) and their heterostructures have attracted significant interest in both academia and industry because of their unusual physical and chemical properties. They offer numerous applications, such as electronic, optoelectronic, and spintronic devices, in addition to energy storage and conversion. Atomic and structural modifications of van der Waals layered materials are required to achieve unique and versatile properties for advanced applications. This review presents a discussion on the atomic-scale and structural modifications of 2D TMDs and their heterostructures *via* post-treatment. Atomic-scale modifications such as vacancy generation, substitutional doping, functionalization and repair of 2D TMDs and structural modifications including phase transitions and construction of heterostructures are discussed. Such modifications on the physical and chemical properties of 2D TMDs enable the development of various advanced applications including electronic and optoelectronic devices, sensing, catalysis, nanogenerators, and memory and neuromorphic devices. Finally, the challenges and prospects of various post-treatment techniques and related future advanced applications are addressed.

## Abstract 2

Atomic and structural modification is an effective method for fine tuning the physical and chemical properties of 2D TMDs and their heterostructures, which enables various advanced applications.

## Graphical abstract

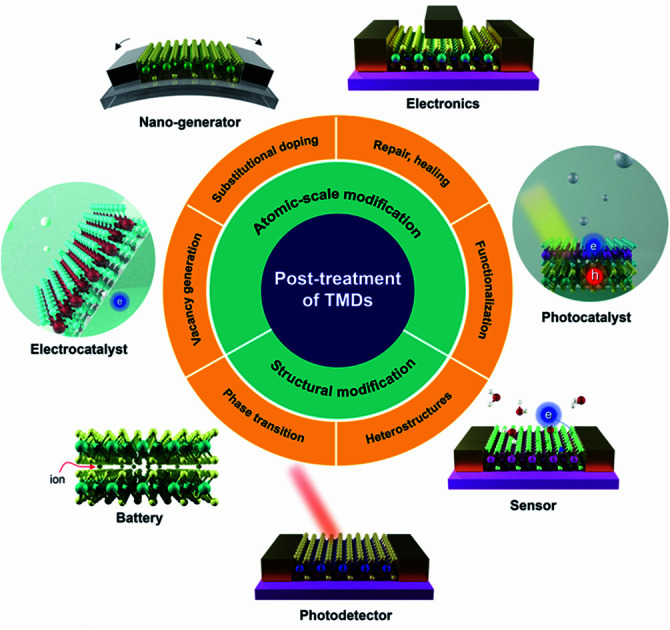

## 1. Introduction

Since the discovery of monolayer graphene (Gr) in 2004,^1^ two-dimensional (2D) transition metal dichalcogenides (TMDs) and their heterostructures have gained significant attention owing to their numerous unique physical and chemical properties, such as high electron mobility,^2^ thermal conductivity,^3^ topological insulation,^4^ Moiré superlattices,^5^ unconventional superconductivity,^6,7^ piezoelectricity,^8,9^ giant magnetoresistance,^10^ non-linear optics,^11–13^ and Weyl semimetals.^14,15^ These unique properties have numerous applications in electronics, optoelectronics, spintronics, valleytronics, energy harvesting, and quantum computation. However, the physical and chemical properties of as-grown 2D TMDs often do not meet the specific requirements for advanced applications.

For example, monolayer molybdenum disulfide flakes grown by chemical vapor deposition (CVD) do not exhibit high carrier mobility in field-effect transistors (FETs) because of intrinsic sulfur vacancies (S_V_) (S_V_ density ≈ 1.24 × 10^13^ cm^−2^), which limits electronic device applications.^16^ This shortcoming can be easily solved by using post-treatment techniques such as thermal annealing under a sulfur-rich atmosphere. Therefore, post-treatment is a promising method of controlling the physicochemical properties of TMDs and, thus, enables the development of various advanced applications.

Although the post-treatment of TMDs and their heterostructures are important for numerous device applications, only specific topics related to defect engineering, phase engineering, and substitutional doping have been reviewed.^17–19^ Furthermore, applications have not been comprehensively discussed in conjunction with post-treatment methods. Therefore, post-treatment methods for TMDs and their heterostructures must be investigated by focusing on atomic-scale and structural modifications along with their device applications.

**Fig. 1:**
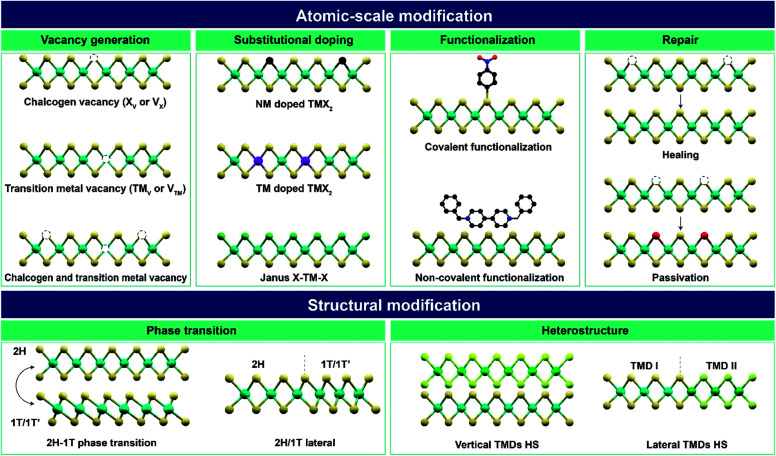
Overview of atomic-scale and structural modifications of 2D TMDs and vdW heterostructures *via* various post-treatment techniques (X – chalcogen; TM – transition metal; V – vacancy; NM – non-metal; HS – heterostructure).

In this review, various post-treatment approaches for the atomic-scale and structural modifications of TMDs are summarized (Fig. 1). Atomic-scale modification is classified into four categories: (I) vacancy generation, (II) substitutional doping, (III) functionalization, and (IV) repair. Structural modification consists of two categories: (V) phase transition and (VI) heterostructures. These modifications modulate the material properties including optical, electronic, catalytic, magnetic properties and so on. Therefore, various advanced applications including electronic and optoelectronic devices, catalysis, energy storage, sensors, piezoelectricity, nanogenerators, and memory and neuromorphic devices according to each post-treatment are discussed. Finally, the challenges and prospects of post-treatment techniques and important issues to be resolved in near future are addressed.

## 2. Atomic-scale modification of TMDs

This section details various atomic-scale modifications (namely, vacancy generation, substitutional doping, functionalization, and repair) of TMDs for device applications. In the first sub-section, vacancy generation methods, including plasma treatment, electron-beam irradiation, thermal annealing, and chemical treatment, are introduced for FETs, electrocatalysts, CO_2_ hydrogenation, and Li–air batteries. The second sub-section describes the substitutional doping of non-metals and metals for applications, such as FETs, biosensors, catalysis, and piezoelectricity. In the third sub-section, covalent and non-covalent functionalizations are discussed for CoVID-19 sensors, triboelectric nanogenerators (TENGs), multifunctional optoelectronic devices, and memory and neuromorphic device applications. The last sub-section describes the repair of atomic defects in TMDs for high-performance flexible piezoelectric nanogenerators (PENGs), photodiodes, superconductors, and low contact resistance in FETs.

### 2.1. Vacancy generation

Vacancy generation in TMDs is highly desirable for controlling the performance of catalytic, electronic, and optoelectronic devices *via* modulating the carrier concentration and tuning the catalytic activity. The focus in most of the previous studies was chalcogen vacancy generation (hereafter, S and Se vacancies are denoted as S_V_ and Se_V_, respectively). Vacancy generation can be categorized into (i) dry etching processes, including plasma treatment, electron/ion-beam irradiation, and thermal annealing, and (ii) wet chemical etching processes.

#### Dry etching process

**Fig. 2:**
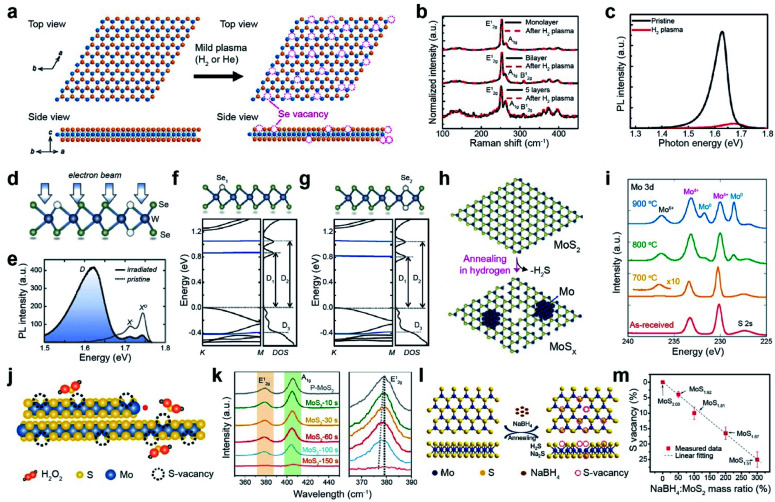
Vacancy generation of 2D TMDs with dry (plasma treatment, electron beam irradiation, and thermal annealing) and wet chemical etching processes. **Plasma treatment:** (a) atomic structure of WSe_2_ layers before and after the H_2_ plasma treatment, illustrating the creation of Se vacancies. (b) Raman spectra of monolayer, bilayer, and five-layer WSe_2_ before and after H_2_ plasma treatment, respectively. (c) PL spectra of the WSe_2_ monolayer before and after H_2_ plasma treatment at room temperature. **Electron beam irradiation:** (d) illustration of the atomic structure of WSe_2_ with a single selenium vacancy (Se_1_) and double selenium vacancy (Se_2_) after electron beam irradiation. (e) PL spectra of the pristine and electron beam irradiated WSe_2_, taken at 5 K. (f and g) DFT band structures of Se_1_ and Se_2_. **Thermal annealing:** (h) schematic illustration of the thermal texturization process of MoS_2_ by thermal annealing under a H_2_ atmosphere. With increasing S_V_ density, a Mo cluster is also generated. (i) XPS spectra of the Mo 3d core level for as-received and annealed bulk MoS_2_ samples. **Chemical etching:** (j) schematic of the chemical etching process with H_2_O_2_. (k) Raman spectra of MoS_2_ with different etching times with H_2_O_2_. Right Raman spectra show a red-shift of the E^1^_2g_ peak according to the etching time. (l) Schematic of the desulfurization process of MoS_2_*via* solid-phase reduction. (m) S_V_ content variation as a function of mass ratio of NaBH_4_ : MoS_2_, obtained from XPS measurement. (a–c) Adapted with permission.^27^ Copyright 2016, American Chemical Society. (d–g) Adapted with permission.^35^ Copyright 2021, American Physical Society. (h and i) Adapted with permission.^51^ Copyright 2016, American Chemical Society. (j and k) Adapted with permission.^52^ Copyright 2020, American Chemical Society. (l and m) Adapted with permission.^54^ Copyright 2019, American Chemical Society.

The dry etching process has the advantage of precisely controlling vacancy density. Plasma consists of electrically charged particles that are produced after the ionization of gases.^20^ Argon plasma, for example, effectively generates defects in TMDs, including MoS_2_,^21–23^ WS_2_,^21^ MoSe_2_,^24^ WSe_2_,^25^ and PtSe_2_.^26^ To prevent severe damage to TMDs, mild plasma treatment with H_2_ or He is employed.^27^ The atomic structure of WSe_2_ after plasma treatment shows S_V_ generation (Fig. 2a). The corresponding Raman spectra of one-, two-, and five-layer WSe_2_ before and after H_2_ plasma treatment display no changes in the peak positions of the characteristic phonon modes (E^1^_2g_ and A_1g_) and their intensities (Fig. 2b), indicating that the crystal lattice of WSe_2_ remains even after the selective removal of Se atoms by plasma. The Se/W ratio of WSe_2_ after plasma treatment is slightly decreased from 2.05 to 1.98 (less than ∼5 at% of Se_V_). The photoluminescence (PL) spectra of the WSe_2_ monolayer after H_2_ plasma treatment are broadened and quenched (Fig. 2c). Oxygen plasma treatment is used for the formation of oxygen-transition metal bonds, such as Mo–O in MoS_2_, Re–O in ReS_2_, and Te–O in WTe_2_ after the removal of chalcogen atoms.^28–31^ In the case of WTe_2_, oxygen plasma can efficiently eliminate Te or W atoms, resulting in the formation of transition metal and chalcogen vacancies, in addition to W–O and Te–O bonds.^32^

Electron beam irradiation can precisely control the generation of chalcogen vacancies *via* several mechanisms such as the knock-on effect, ionization, beam-induced chemical etching, and ballistic displacement.^33,34^ The atomic structure of electron-beam-irradiated WSe_2_ presents both single (Se_1_) and double selenium vacancies (Se_2_) (Fig. 2d).^35^ The neutral (X^0^) and negatively charged (X^−^) exciton peaks of pristine WSe_2_ are observed in its PL spectra. The additional broad defect band (D) with attenuated X^0^ and X^−^ intensities are observed for irradiated WSe_2_ by performing scanning electron microscopy (SEM) with ∼10^7^ electrons per μm^2^ (Fig. 2e). The density functional theory (DFT) band structures of Se_1_ and Se_2_ represent the formation of unoccupied midgap bands (D_1_ and D_2_), in addition to the occupied one below the valence-band maximum (D_3_) (Fig. 2f and g).^35^ The number of chalcogen vacancies in MoS_2_ and MoSe_2_ can be precisely controlled by an electron beam in *in situ* transmission electron microscopy (TEM).^36,37^ In addition to electron beams, ion beams such as argon,^38,39^ helium,^40–42^ manganese,^43^ gallium,^44,45^ and gold,^46^ can be employed to generate vacancies. For example, the S_V_ content in MoS_2_ and WS_2_ monolayers gradually increases with Ar^+^ ion beam irradiation.^47–50^

Thermal annealing is a simple method for creating chalcogen vacancies *via* thermal desorption. A H_2_ atmosphere is commonly used during thermal annealing to etch away chalcogen atoms *via* H_2_S and H_2_Se formation (Fig. 2h). The thermal texturization process of MoS_2_ flakes is also observed.^51^ The XPS spectra of the Mo 3d core level for MoS_2_ after annealing at elevated temperatures (700, 800, and 900 °C) shows the gradual evolution of peaks at 232 and 229 eV (assigned to the Mo^0^ doublet) and 236.5 eV (assigned to Mo^6+^), in addition to a doublet at 233 and 230 eV (assigned to Mo^4+^ 3d_3/2_ and 3d_5/2_, respectively) for pristine MoS_2_ (Fig. 2i). Such evolution of a Mo^0^ doublet and Mo^6+^ peaks is attributed to the formation of Mo metal clusters (by the removal of S atoms) and MoO_3_ at S_V_ sites (due to Mo oxidation under air), respectively. In contrast, the S 2p doublet in the S 2p core-level spectra is still present, indicating that some MoS_2_ can be maintained, regardless of the annealing temperature.

#### Wet chemical etching process

Wet chemical etching is a facile and mild strategy for creating atomic chalcogen and transition metal vacancies. Various reagents, such as H_2_O_2_,^52,53^ NaBH_4_,^54–56^ hydrazine,^57^ and HCl,^58^ have been utilized. For example, MoS_2_ nanosheets are immersed in a H_2_O_2_ solution to generate vacancies (Fig. 2j). The Raman spectra of the MoS_2_ nanosheets with H_2_O_2_ treatment show that both the E^1^_2g_ and A_1g_ peaks gradually broaden and eventually disappear, indicating that the S and Mo atoms are progressively etched away over the treatment time (Fig. 2k).^52^ Phonon softening related to the redshift of the E^1^_2g_ peak is also observed. The significant intensity changes in the E^1^_2g_ and A_1g_ peaks are attributed to a high vacancy generation of ∼15 at% after chemical etching for 150 s. NaBH_4_ is used for solid-phase reduction to create S_V_ in the basal plane of MoS_2_ (Fig. 2l).^54^ NaBH_4_ exclusively reduces Mo^4+^ to Mo^δ+^ (*δ* < 4), resulting in the removal of sulfur atoms by forming H_2_S and Na_2_S. The concentration of S_V_-MoS_2_ increases almost linearly with the mass ratio of NaBH_4_ : MoS_2_, indicating that the concentration of vacancies can be efficiently controlled by the amount of reagent (Fig. 2m). Hydrazine is used to donate electrons to WS_2_, in addition to creating S_V_.^57^

Although various types of dry etching processes (*i.e.* plasma treatment, electron/ion beam irradiation, and thermal annealing) are employed for vacancy engineering, the detailed mechanisms of vacancy generation are still not well-understood. Plasma or ion beams are more desirable for patterned device applications, whereas thermal annealing is preferable for catalytic applications. Chemical wet etching frequently induces the formation of undesired vacancies and oxides.

#### Applications

**Fig. 3:**
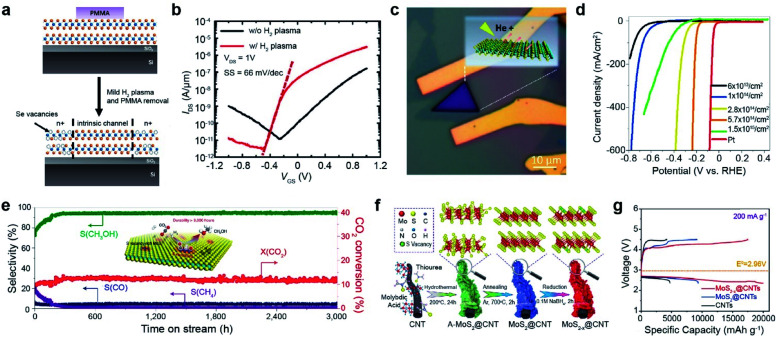
Vacancy-driven applications: FET, electrocatalyst for hydrogen evolution, CO_2_ hydrogenation catalyst, and Li–air battery. **FET:** (a) schematic of selective vacancy generation with H_2_ plasma treatment and (b) corresponding *I*_DS_–*V*_GS_ curves of the WSe_2_ FET with/without plasma treatment, measured under 1 × 10^−5^ Torr. **Electrocatalyst for hydrogen evolution:** (c) optical image of He^+^ ion beam irradiated MoS_2_. (d) LSV curves of MoS_2_ at various S_V_ concentrations and Pt wire. **CO**_**2**_**hydrogenation catalyst:** (e) long term stability test of the S_V_-MoS_2_ nanosheet catalyst in the hydrogenation of CO_2_ at 3000 mL g_cat._^−1^ h^−1^, measuring the selectivity (*S*) of CH_3_OH, CO, and CH_4_ and conversion (*X*) of CO_2_ over 3000 h. **Li–air battery:** (f) synthesis process of MoS_2−*x*_@CNTs. (g) Charge/discharge profiles at 200 mA g^−1^ in the voltage range from 2.35 to 4.5 V. (a and b) Adapted with permission.^27^ Copyright 2016, American Chemical Society. (c and d) Adapted with permission.^68^ Copyright 2019, American Chemical Society. (e) Adapted with permission.^73^ Copyright 2021, Springer Nature Limited. (f and g) Adapted with permission.^76^ Copyright 2022, Wiley-VCH.

The vacancies in TMDs can be utilized for various applications, including FETs, electrocatalytic microcells for hydrogen evolution, CO_2_ hydrogenation catalysts, and Li–air batteries. Anion vacancies, such as S_V_ and Se_V_, serve as n-type dopants. Thus, the electrical conductivity of TMDs increases with the vacancy concentration until a certain point and then decreases at high concentrations because of the collapse of the crystal structure.^27,59–61^ The optimized concentration of Se_V_ minimized contact resistance by forming edge contacts in a homojunction (Fig. 3a). Mild H_2_ plasma was introduced to selectively generate Se_V_ in the contact region in the WSe_2_ FET. As a result, the on-current was significantly increased by a factor of 20, attaining a low subthreshold swing (SS) of 66 mV dec^−1^ (Fig. 3b).^27^ Furthermore, semiconductor-to-metallic transitions in MoS_2_ and WS_2_ were also observed with low-energy He plasma and He^+^ beam irradiation treatments.^61–63^ This transition was attributed to the emergence of mid-gap states near the Fermi level at an appropriate vacancy concentration.

The edge of 2H-MoS_2_ acts as an active site for hydrogen evolution, whereas the basal plane is inactive.^64^ In addition, low electrical conductivity limits the electrocatalytic activity of MoS_2_. Vacancy generation in MoS_2_ is very useful for activating its basal plane for the hydrogen evolution reaction (HER) and increasing its electrical conductivity.^65–67^ For example, the basal plane of MoS_2_ was irradiated with a He^+^ beam (Fig. 3c). Consequently, with increasing S_V_ concentration, the linear sweep voltammetry (LSV) curves shifted toward the Pt reference up to an optimum concentration of 5.7 cm^14^ cm^−2^,^68^ which is clear evidence of the activation of the basal plane by vacancy generation. When the S_V_ concentration was increased above this threshold, the LSV curves shifted backward (Fig. 3d), attributed to the collapse of the MoS_2_ structure. Many approaches, including NaClO treatment,^69^ ozone treatment,^70^ and laser treatment,^71^ have been used for vacancy generation to enhance the HER activity of TMDs. In quantum information applications, single-photon emission from the defects of the WSe_2_ monolayer induced by e-beam irradiation has recently been reported.^72^

The basal plane of S_V_-MoS_2_ is an ideal active site for low-temperature hydrogenation of CO_2_ to selectively produce methanol *via* the following reaction mechanism: (i) dissociation of CO_2_ to surface-bound CO* and O* at S_V_ sites, (ii) hydrogenation of CO* to CH_3_O*, and (iii) synthesis of CH_3_OH (inset of Fig. 3e). As a result, S_V_-rich MoS_2_ nanosheet catalysts showed a high methanol selectivity of 94.3% and CO_2_ conversion of 12.5%. Furthermore, they were stable over 3000 h at 180 °C (Fig. 3e).^73^ S_V_ in MoS_2_ nanoflowers prepared by thermal annealing in a hydrogen environment also improves solar-driven CO_2_ photoreduction.^74^ The rate of CO production was enhanced approximately 2-fold after S_V_ generation.

Chalcogen vacancies provide abundant active sites for the intercalation/deintercalation of guest ions (Li^+^, Na^+^, and K^+^), which leads to enhanced reaction kinetics and improved specific capacities. For example, sulfur vacancies in MoS_2_ can intrinsically promote O_2_ adsorption, enhancing the electrochemical performance of Li–O_2_ batteries.^75^ The core–shell MoS_2−*x*_@CNT composite synthesized by hydrothermal and thermal annealing was treated with NaBH_4_ to increase S_V_ concentration (Fig. 3f). The initial discharge/charge profile at 200 mA g^−1^ was significantly boosted up to discharge/charge specific capacities of 19 989/17 705 mA h g^−1^ with an overpotential of 0.99/0.26 V (Fig. 3g).^76^ Furthermore, S_V_ improves polysulfide conversion kinetics in Li–S batteries,^77^ facilitates the absorption of Na^+^/Zn^+^, and increases the conductivity of Na/Zn-ion batteries.^78,79^

### 2.2. Substitutional doping

The substitution doping of TMDs is classified into two main categories: impurity doping with partial substitution and Janus structures with full replacement of chalcogen atoms of the top layer (out of three atomic layers) in TMDs. Impurity doping is an important technique for precisely controlling the electrical and other intrinsic properties of TMDs for next-generation high-end electronics, optoelectronics, medicine, and energy harvesting applications.^80,81^ Several approaches have been developed to tune the characteristics of TMDs by substituting chalcogen atoms (X) or transition metals (M) *via* atomic doping.^82,83^ Depending on the relative valency of the dopant atoms, they act as electron donors or acceptors. When a Janus group-VI chalcogenide MXY (top layer X, bottom layer Y = S, Se, and Te; X ≠ Y) is formed, the out-of-plane mirror symmetry is broken. This unique asymmetrical feature of Janus structures arises from different atomic radii and electronegativities of X and Y atoms, thus enabling novel applications such as piezoelectric devices and electrocatalysts.^84,85^

#### Impurity doping

**Fig. 4:**
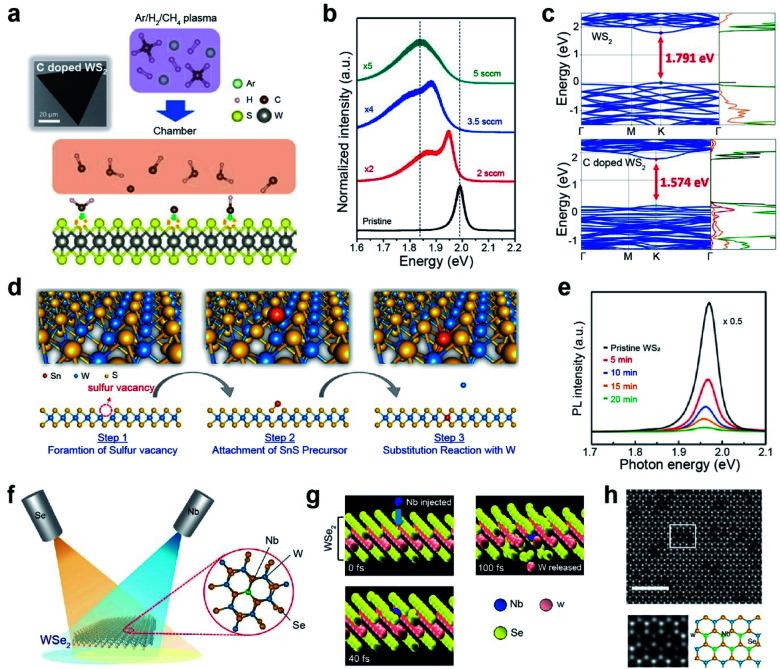
Non-metallic and metallic impurity doping in TMDs: plasma-assisted, thermal annealing, and beam epitaxy**. Non-metallic doping:** (a) schematic of the plasma-assisted doping experiment (right) and SEM image (left) of the carbon-doped monolayer WS_2_. (b) PL spectra of the pristine and carbon-doped monolayer WS_2_ with different methane flow rates (2, 3.5, and 5 sccm, respectively). (c) Band structure and density of states (DOS) of monolayer pristine WS_2_ and CH-doped WS_2_. **Transition metal doping:** (d) doping mechanism of Sn substituting into the W site in the WS_2_ layers. (e) PL spectrum of monolayer WS_2_ before and after 5, 10, 15, and 20 min of Sn doping. (f) Schematic representation of Nb doping in WSe_2_ with a dopant (Nb) and a chalcogen (Se) beam. (g) Snapshots of the Nb substitutional doping process in WSe_2_ after 0, 40, and 100 fs, respectively, obtained by *ab initio* molecular dynamics simulation. (h) Representative HAADF-STEM image of monolayer WSe_2_ after doping of Nb. The scale bar corresponds to 2 nm. Cut out from the white squares is matched with the structural model as shown. In the structural model, blue, green, and yellow balls represent W, Nb, and Se atoms, respectively. (a–c) Adapted with permission.^95^ Copyright 2019, American Association for Advancement of Science. (d and e) Adapted with permission.^101^ Copyright 2019, American Chemical Society. (f–h) Adapted with permission.^102^ Copyright 2021, American Chemical Society.

Non-metallic (NM) doping of TMDs has been performed by substituting chalcogen atoms with O, Te, Cl, N, P, and F atoms.^86–94^ For example, carbon doping was carried out *via* plasma-induced CH_4_ gas exposure of WS_2_ monolayers (Fig. 4a).^95^ No noticeable damage was observed in the monolayers, and their PL intensity progressively decreased and shifted toward lower energy values with increasing carbon content (Fig. 4b). Theoretical simulations predicted that, unlike single C doping, CH doping provided the most stable and lowest local stain on WS_2_. The calculated bandgap of pristine WS_2_ was 1.791 eV, whereas that of carbon-doped WS_2_ had a direct bandgap of 1.574 eV (Fig. 4c). After carbon doping, the energy gap is decreased by raising new acceptor levels above the valence band of WS_2_ owing to the hybridization of W d-orbitals and C p-orbitals. The extra holes can move to new levels, implying that CH impurities act as p-type dopants.

Transition metal (TM) doping at the M site of a TMD is conducted in three steps: (i) generation of chalcogen vacancies, (ii) replacement of transition metal-adjacent chalcogen vacancy sites, and (iii) healing of chalcogen vacancies.^96–100^ For example, WS_2_ monolayers were first grown with S_V_ sites, followed by subsequent exposure to a Sn-rich atmosphere using SnS as a precursor at 550 °C (Fig. 4d).^101^ The characteristic PL peak of WS_2_ was gradually attenuated with doping time and dopant concentration (Fig. 4e). Moreover, Sn dopants in the WS_2_ lattice act as electron donors (*i.e.*, n-type dopants). Another strategy for TM doping is the direct use of a metal flux with the aid of electron beam evaporation (Fig. 4f).^97,102^ In this case, dopant beams (such as Nb and Re) with low kinetic energy are generated by thermal evaporation of high-melting-point metals on an exposed TMD. The beam flux is modulated to supply metal dopants during the entire doping process to enhance structural reconstruction and regulate the formation of metal-doped TMDs. Concurrently, a Se beam is continuously supplied to heal possible Se vacancies during the doping process. Accurate and position-selective doping can be achieved when patterned TMD materials are used.^102^ Molecular dynamics (MD) simulations showed a gradual structural change with Nb and Se exposure (Fig. 4g). When a Nb atom hit a W atom with substantial energy (simulation time is varied from 0 to 100 fs), the W atom was released from the original position. Simultaneously, the Nb atom replaced the vacancy created by the released W atom. Fig. 4h displays an atomic-resolution high-angle annular dark-field (HAADF)-scanning transmission electron microscopy (STEM) image of monolayer WSe_2_ after exposure to a Nb beam. The cutouts from the white squares in Fig. 4h clearly show the atomic structure of Nb-doped WSe_2_: bright and dark spots at W sites in the hexagonal lattice are assigned to W and Nb atoms (as shown in the structural model). A summary of impurity doping in CVD-grown monolayers and exfoliated TMDs is presented in Table 1.

**Table 1:** Impurity doping of CVD-grown and exfoliated monolayer TMDs with transition metal (TM), non-metal (NM), chalcogen (X), and halogen (H) atoms

Category	Doping sites	Dopants	Host	Type	Doping method	Application	References
X	X site	Te	MoS_2_, WS_2_	p	NaOH assisted Te deposition	—	87
X	X site	Te	MoS_2_	—	Te vapor deposition	—	88
H	X site	Cl	MoS_2_, MoSe_2_	n	Cl ion implantation	Electronics	89
NM	X site	N	MoS_2_, WS_2_	p	N_2_ plasma treatment	Electronics	91
NM	X site	P	MoS_2_, WS_2_	p	PH_3_ plasma treatment	Electronics	93
H	X site	F	MoS_2_	p	SF_6_ plasma treatment	Electronics	94
NM	X site	C	WS_2_	p	CH_4_ plasma treatment	FET	95
TM	M site	V	MoTe_2_	p	Vapor	Ferromagnetism	96
TM	M site	Nb	WSe_2_	p	Molecular beam epitaxy	—	97
TM	M site	Nb, Co	WSe_2_	—	Physical ion implantation	Ferromagnetism	98
TM	M site	Co	WSe_2_	—	Physical ion implantation	Ferromagnetism	99
TM	M site	Mo/Ti	MoS_2_, MoTe_2_	—	Mo/Ti diffusion	—	100
TM	M site	Sn	WS_2_	n	SnS vapor	Electronics	101

#### Janus structures

Janus TMD structures can be obtained using two representative methods: (i) thermal annealing under a chalcogen-rich atmosphere and (ii) H_2_ plasma stripping and thermal chalcogenization at low temperature (hereafter denoted as remote plasma-assisted chalcogenization).^103–106^

In the past, Janus structures were constructed by a thermal-annealing process under a secondary chalcogen atmosphere. For example, Janus MoSSe and MoSeS structures were produced by annealing MoSe_2_ and MoS_2_ at temperatures of ∼800 and ∼450 °C under S and Se atmospheres, respectively.^103,104^ However, high temperatures are unfavorable for 2D Janus monolayers because of a high probability of alloy or defect formation, such as chalcogen vacancies and cracks.

**Fig. 5:**
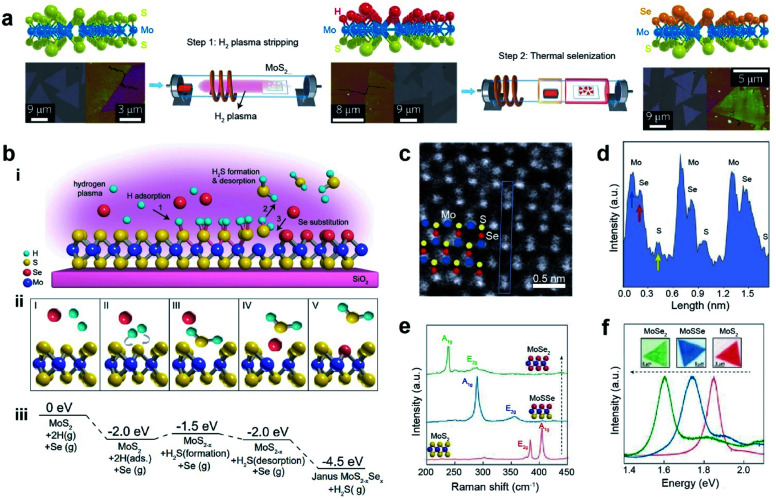
Janus formation *via* remote plasma-assisted chalcogenization. (a) Atomic structure (top), optical (bottom left), and AFM (bottom right) images of CVD-grown MoS_2_, hydrogenated MoS_2_, and Janus MoSSe_2_ monolayer after two steps of H_2_ plasma stripping and thermal selenization. (b) (i) Schematic of the room temperature atomic layer substitution (RT-ALS) process of monolayer MoS_2_. (ii) Schematic of the five key reaction steps in the RT-ALS process (cartoons from left to right): (I) before H adsorption, (II) two H atom adsorption and diffusion to the same S, (III) formation of H_2_S, (IV) desorption of H_2_S, and (V) Se occupation of the S vacancy. Purple, yellow, red, and green balls are Mo, S, Se, and H atoms, respectively. (iii) Free energy of each step in B, relative to that of the first step. (c) Tilted STEM image of MoSSe (seen from a vertical direction). Corresponding Mo, Se, and S atoms are shown with blue, red, and yellow circles, respectively. (d) Intensity profile for the atomic structure in the blue box in (c) shows the different intensities of individual Mo, Se, and S atoms. (e and f) Raman and PL spectra of pristine monolayer MoS_2_, Janus MoSSe, and fully converted MoSe_2_ respectively. (a) Adapted with permission.^103^ Copyright 2017, Springer Nature Limited. (b–f) Adapted with permission.^107^ Copyright 2021, National Academy of Sciences.

Remote plasma-assisted chalcogenization consists of two consecutive steps: (i) remote hydrogen plasma treatment of a CVD-grown MoS_2_ monolayer to strip off sulfur atoms from the top layer and replace them with hydrogen atoms and (ii) replacement of H atoms with Se through a thermal selenization process at ∼450 °C to form a structurally stable Janus MoSSe monolayer (Mo atoms are covalently bonded to the underlying S and top-layer Se atoms) (Fig. 5a).^103^ Similarly, a room-temperature atomic layer substitution (RT-ALS) process was recently developed (Fig. 5b–i).^107^ This less disruptive technique employs hydrogen radicals produced by a remote plasma to strip chalcogen atoms on the top layer of an as-grown TMD. Concurrently, vaporized chalcogens (Se or S) are supplied to substitute stripped atoms, resulting in an asymmetric Janus structure at room temperature in the form of MXY (M = Mo or W, X = S or Se, and Y = Se or S). Fig. 5b(ii) depicts the formation of a Janus structure: (I) before H and chalcogen adsorption, (II) adsorption and diffusion of two H atoms to the same S, (III) formation of H_2_S, (IV) desorption of H_2_S, and (V) Se occupation of the S vacancy. The free energy values for each step are shown in Fig. 5b(iii). The tilted ADF-STEM image reveals that Se atoms are located on one side of the monolayer MoSSe, while S atoms are on the opposite side. This is direct evidence of a Janus structure (Fig. 5c). The corresponding intensity profile in Fig. 5d clearly shows individual Mo, Se, and S atoms with peak intensities proportional to their atomic numbers. Furthermore, the MoS_2_ A_1g_ (404 cm^−1^) and E_2g_ (383 cm^−1^) modes in the Raman spectra shifted to 288 cm^−1^ and 355 cm^−1^, respectively, due to disrupted symmetry in the vertical direction caused by the formation of a Janus structure (Fig. 5e). After further selenization processing of Janus MoSSe was performed, a sharp peak at ∼239 cm^−1^ and a broad peak at ∼284 cm^−1^ were formed, which are the signature peaks of the A_1g_ and E_2g_ modes in monolayer MoSe_2_. In addition, the PL shifts from 1.85 eV (pristine MoS_2_) to 1.72 eV (Janus MoSSe) and finally to 1.60 eV (converted MoSe_2_) are clearly observed (Fig. 5f).^103,105^

Room-temperature doping with remote plasma-assisted chalcogenization offers the possibility of producing high-quality TMDs and 2D Janus structures, which will advance the fabrication techniques for industrial applications as a desirable emerging platform.

#### Applications

The tunable material properties obtained by substitutional doping of TMDs have been utilized to realize novel applications, including electronics, biosensors, catalysis, optoelectronic applications, magnetization, and photocatalysis.

**Fig. 6:**
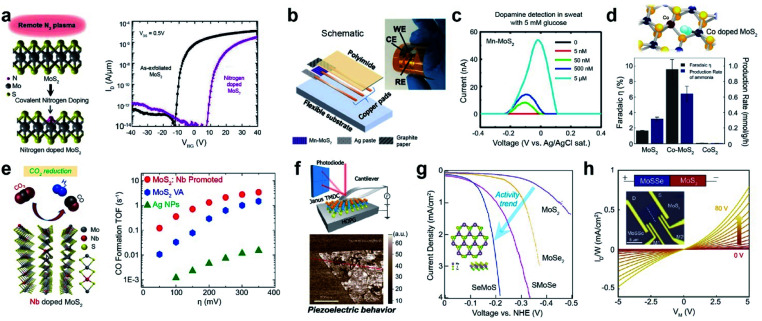
Applications of substitutionally doped TMDs: electronic, biosensor, catalysis, and piezoelectricity. **Electronic:** (a) schematic of covalent nitrogen doping in MoS_2_ upon N_2_ plasma surface treatment (left) and *I*_DS_–*V*_GS_ characteristics of a multilayer nitrogen-doped MoS_2_ FET (right). **Biosensor:** (b) assembled integrated sensor for detecting dopamine (DA) levels. (c) Differential pulse voltammetry results with DA in biologically complex samples (artificial sweat containing 5 mM glucose). **Nitrogen and CO**_**2**_**reduction catalysis:** (d) NRR performance of MoS_2−*x*_, Co-doped MoS_2−*x*_, and CoS_2_ at 0.3 V overpotential. Co doping of MoS_2−*x*_ dramatically increases its faradaic efficiency. CoS_2_ is inactive for the NRR. (e) Schematic representation of Nb-MoS_2_ and reduction of CO_2_ (left), in addition to the calculated CO formation TOF at different applied potentials for Ag NPs, VA-MoS_2_, and VA-Mo_0.95_Nb_0.05_S_2_ in a CO_2_ environment (right). **Piezoelectricity:** (f) schematics and isolated Janus MoSSe monolayers measured by resonance-enhanced piezoresponse force microscopy. HER electrocatalyst: (g) HER polarization curves of monolayer MoS_2_, MoSe_2_, SMoSe, and SeMoS. Electronic: (h) four-probe output characteristics (current density (*I*_D_/*W*) *versus* bias voltage (*V*_M_)) with various back-gate voltages (*V*_G_) for MoSSe–MoS_2_. (a) Reproduced with permission.^91^ Copyright 2016, American Chemical Society. (b and c) Reproduced with permission.^108^ Copyright 2020, the American Association for Advancement of Science. (d) Reproduced with permission.^111^ Copyright 2019, American Chemical Society. (e) Reproduced with permission.^112^ Copyright 2019, American Chemical Society. (f) Reproduced with permission.^103^ Copyright 2017, Springer Nature Limited. (g) Reproduced with permission.^104^ Copyright 2017, American Chemical Society. (h) Reproduced with permission.^107^ Copyright 2021, National Academy of Sciences.

Impurity doping is widely used to modulate the electrical properties of TMD materials.^91^ For example, the *I*–*V* transfer curve of n-type-doped multilayer MoS_2_ obtained by N_2_ plasma exposure distinctly shows a positive threshold voltage (*V*_th_) shift, which is consistent with the p-type dopant behavior of nitrogen in MoS_2_ (Fig. 6a). Moreover, Mn-doped MoS_2_ (Mn-MoS_2_) was used to selectively detect dopamine (DA) levels in serum and artificial sweat.^108^ Abnormal levels of dopamine in the body can be symptomatic of several disorders such as Alzheimer's disease, schizophrenia, and Parkinson's disease.^109^ Previously, DA detection was achieved by employing highly sophisticated methods, such as mass spectrometry, liquid chromatography, and electrochemical detection measurements.^110^ Therefore, a low-cost but accurate diagnostic tool for the detection of DA levels is essential. A wearable DA sensor was fabricated on a flexible polyimide (PI) sheet with a Mn-MoS_2_ working electrode (WE), a pyrolytic graphite sheet (PGS) counter electrode (CE), and an Ag paste reference electrode (RE) (Fig. 6b). DA concentrations as low as 50 nM were successfully detected in artificial sweat containing 5 mM glucose (Fig. 6c). Furthermore, Co-doped defective MoS_2_ (Co-MoS_2_) exhibits superior dinitrogen-to-ammonia conversion activity compared with pristine MoS_2_ and CoS_2_ (Fig. 6d).^111^ Such a high faradaic efficiency and production rate are attributed to the effective activation of the dinitrogen molecule for the dissociation of the N≡N triple bond in defective MoS_2−*x*_.

The electrocatalytic conversion of CO_2_ into sustainable fuels is considered the most efficient approach for achieving carbon neutrality.^112^ Nb-doped MoS_2_ can reduce CO_2_ to produce useful hydrocarbon derivatives, such as methane and ethanol, along with H_2_ (Fig. 6e). The CO formation turnover frequency (TOF) of Nb-doped MoS_2_ in an ionic liquid is one order of magnitude higher than that of Ta-doped MoS_2_ or Ag NPs in the overpotential range of 50–150 mV. Furthermore, the current density of Nb-doped MoS_2_ in LSV experiments was approximately 2 and 50 times higher than that of pristine MoS_2_ and Ag NPs, respectively.

The Janus structure of TMDs has been reported to possess piezoelectric properties due to its non-symmetrical structure, which generates electrical polarization in response to externally applied mechanical stress.^103^ The resonance-enhanced piezoresponse force microscopy image of Janus MoSSe shows the presence of piezoelectric properties (Fig. 6f), which are not observed in pristine MoS_2_. In addition, Janus TMD monolayers effectively activate TMD basal planes for the HER.^104^ Janus SMoSe (for MoSSe) and its reverse configuration of SeMoS (for MoSeS) were constructed by atomic substitution of pure MoS_2_ and MoSe_2_, respectively (Fig. 6g). Both Janus SMoSe and SeMoS monolayers exhibit lower overpotentials in the LSV curves than pure MoS_2_ and MoSe_2_. Moreover, the HER activity of SeMoS surpasses that of SMoSe, which is attributed to the greater thermoneutral Gibbs free energy (Δ*G*_H_) for SeMoS in the presence of Se_V_ (−0.007 eV for SeMoS and 0.060 eV for SMoSe). The unique structure of Janus TMDs enables band edge modulation and electronic transfer when heterostructures are designed. Taking advantage of the type-II band alignment of MoSSe–MoS_2_, a back-gate transistor was fabricated to measure the four-probe output characteristics (Fig. 6h).^107^ A plot of current density (*I*_D_/*W*) *versus* bias voltage (*V*_M_) at various applied back-gate voltages (*V*_G_) shows weak rectification behavior at the lateral junction.

### 2.3. Functionalization

Functionalization leads to improvement in the optical, electronic, and sensing characteristics of TMDs for next-generation devices. Functionalization with organic molecules is categorized into (i) covalent functionalization *via* chemisorption and (ii) non-covalent functionalization *via* physisorption.^113,114^ Functional groups in an organic molecule effectively tune the Fermi level of TMDs *via* an electron-donating or electron-withdrawing mechanism. Hydroxyl (–OH), alkoxyl (–OR), and amine groups (–NH_2_) donate electrons (*i.e.*, they are n-type dopants) to TMDs, while nitro (–NO_2_), cyano (–CN), and tri-halogenated methyl (–CX_3_) groups withdraw electrons (*i.e.*, they are p-type dopants) from TMDs.^115–118^

#### Covalent functionalization

Covalent functionalization involves a chemical reaction to form covalent bonds between TMDs and organic molecules. Representative approaches include direct functionalization of metallic TMDs, functionalization of aryl diazonium salts, and functionalization of thiol derivative molecules.

**Fig. 7:**
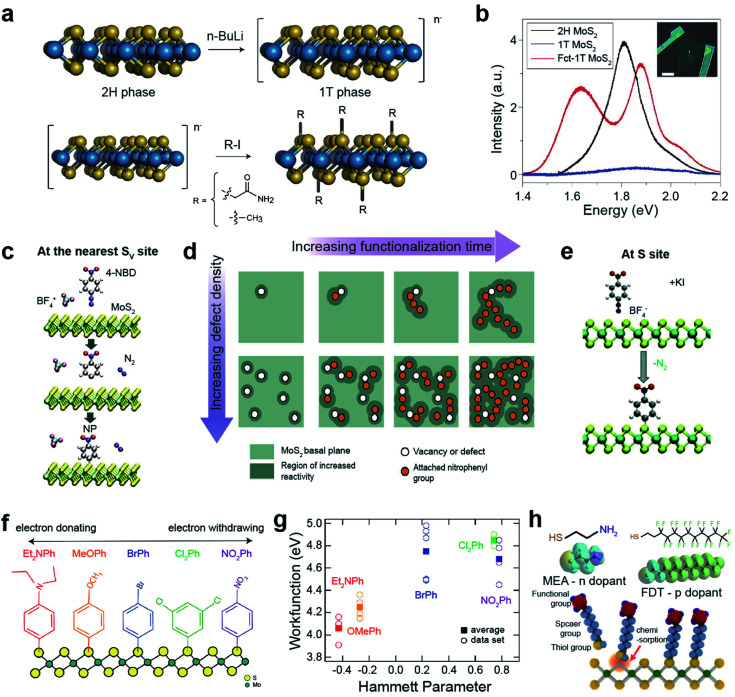
Covalent functionalization of 2D TMDs: direct functionalization on metallic TMDs, aryl diazonium salt functionalization, and thiol derivative molecule functionalization. **Direct functionalization on metallic TMDs:** (a) schematic of the conversion of 2H-MoS_2_ to a negatively charged 1T phase *via* phase transition using *n*-butyl lithium (*n*-BuLi) (top) and subsequent covalent functionalization with alkyl halides (2-iodoacetamide and iodomethane) (bottom). (b) PL spectra of 2H-MoS_2_, 1T-MoS_2_ and functionalized MoS_2_ (Fct-1T-MoS_2_). **Aryl diazonium salt functionalization:** (c) schematic representation of MoS_2_ functionalized by 4-nitrobenzene diazonium (4-NBD) tetrafluoroborate (BF_4_) *via* reduction of a diazonium salt, resulting in a nitrophenyl (NP) radical grafting. (d) Schematic illustration of the reaction mechanism of MoS_2_ with 4-NBD as a function of defect concentration and time. (e) Schematic representation of the direct functionalization of MoS_2_ with 4-nitrobenzene diazonium (4-NBD) using KI activation. (f) Schematic representation of functionalized 1T′-MoS_2_ with various diazonium salts including different functional groups (NO_2_Ph, Cl_2_Ph, BrPh, MeOPh, and Et_2_NPh). (g) Work function of functionalized MoS_2_ with various diazonium salts as a function of the functional group Hammett parameter. **Thiol derivative molecule functionalization:** (h) schematic representation of the chemisorption of thiol molecules onto S_V_-MoS_2_ and the molecular structures of mercaptoethylamine (MEA) and perfluorodecanethiol (FPT). (a and b) Adapted with permission.^119^ Copyright 2015, Springer Nature Limited. (c and d) Adapted with permission.^120^ Copyright 2018, American Chemical Society. (e) Adapted with permission.^123^ Copyright 2021, Royal Society of Chemistry. (f and g) Adapted with permission.^125^ Copyright 2018, American Chemical Society. (h) Adapted with permission.^130^ Copyright 2015, American Chemical Society.

Semiconducting 2H-TMDs, such as MoS_2_, WS_2_, and MoSe_2_, are chemically unreactive because they are free from dangling bonds on their surfaces. However, after the phase transition from 2H to negatively charged metallic 1T-TMDs *via* lithiation, they become reactive to covalent functionalizations with alkyl halides and diazonium salts (Fig. 7a).^119^ For example, the MoS_2_ exciton peak in the PL spectra almost completely disappeared after the phase transition (Fig. 7b) and two prominent peaks evolved in functionalized 1T-MoS_2_ (Fct-1T-MoS_2_). The peak at ∼1.6 eV may be attributed to the band structure modification by covalent functionalization, while the peak at ∼1.9 eV is related to the up-shift of the MoS_2_ exciton emission.^119^ The appearance of PL peaks indicates that the metallic 1T phase is converted to the semiconducting 1T one after functionalization. This was further confirmed by the characteristics of the fabricated field-effect transistor.

Aryl diazonium functionalization can occur at the nearest S_V_ sites of MoS_2_. S_V_ sites, where charge is accumulated, reduce 4-nitrobenzenediazonium tetrafluoroborate (4-NBD), resulting in nitrophenyl (NP) radical grafting after N_2_ release (Fig. 7c).^120^ Localized charge density adjacent to the grafting sites is renormalized after functionalization, further triggering a chain-like growth propagation of subsequent NP molecules over MoS_2_ sulfur sites (top panels in Fig. 7d). A higher S_V_ density promotes numerous initiation reaction sites for the propagation of NP molecules over the entire MoS_2_ basal plane (bottom panels in Fig. 7d). The semiconducting nature of MoS_2_ is still maintained after functionalization. The NP functionalization of S_V_-MoS_2_ obeys pseudo-second-order (adsorbate–surface and adsorbate–adsorbate interactions) reaction kinetics,^121^ whereas the functionalization of Gr obeys first-order (adsorbate–surface interactions) reaction kinetics.^122^ On the other hand, S sites in MoS_2_ can be directly functionalized with NP radicals *via* active chemical reduction of 4-NBD ions, using potassium iodide as an electron donor (Fig. 7e).^123^ Similarly, alkyl halide-functionalized 1T′-MoS_2_ is activated using metallocene reducing agents, which facilitate high surface coverage with alkyl halide groups.^124^ In addition, n-type doping with KI (reducing agent) itself has been observed, providing advantages for low contact resistance and increased charge carrier mobility for FET devices.^123^

One of the advantages of diazonium salts, in terms of functionalization, is the controllability of the terminated molecules, which enables the engineering of the MoS_2_ electronic structure. 1T-MoS_2_ readily reacts with various diazonium salts, including different functional groups (NO_2_Ph, Cl_2_Ph, BrPh, MeOPh, and Et_2_NPh) (Fig. 7f).^125^ Each functional group has a particular Hammett parameter (Fig. 7g). The measured work function of the functionalized MoS_2_ increased almost linearly with the Hammett parameter, indicating that the work function of MoS_2_ can be controlled by functional groups. This surface energy change strongly influences HER catalytic activity. In fact, HER performance is significantly improved by electron-donating Et_2_NPh groups.^125^ Furthermore, covalent functionalization of 1T′-MoS_2_ with Et_2_NPh improves the stability of electronic transport properties for at least two weeks under atmospheric conditions.^126^

Another approach is the selective functionalization of TMD chalcogen vacancies or edge sites with thiol derivative molecules *via* a conjugation reaction.^127–129^ MoS_2_ can be functionalized with thiol molecules (2-mercaptoethylamine, MEA, and 1*H*,1*H*,2*H*,2*H*-perfluorodecanethiol, FDT) at S_V_ sites by soaking for 72 h (Fig. 7f). The charge density of functionalized MoS_2_ was strongly affected by the terminated molecules. NH_2_ groups in MEA donate electrons to MoS_2_ (charge density from 9.4 × 10^11^ to 1.4 × 10^12^ cm^−2^), whereas F groups in FDT withdraw electrons from MoS_2_ (charge density −7.0 × 10^11^ to −1.8 × 10^11^ cm^−2^).^130^ Likewise, functionalization with aromatic or alkyl thiols tunes the electronic structure of MoS_2_. For example, in the case of alkyl thiols, the Fermi energy level (*E*_F_) upshifts with increasing chain length, such as for 1-propanethiol, 1-nonanethiol, and 1-dodecanethiol.^131^

Phase transition requires harsh chemical treatment of TMDs with a highly pyrophoric compound (*n*-butyllithium), which deteriorates the quality of the material.^132,133^ The diazonium salt functionalization approach is commonly used to engineer the electronic structure of TMDs. Thiol-derivative functionalization with TMDs is inherently limited by the number of vacancy sites.

#### Non-covalent functionalization

Non-covalent functionalization consists of the physisorption of organic molecules on the surface of TMDs/Gr without chemical bond formation.^134–136^ This enables chemical doping and formation of heterojunctions on TMDs.

**Fig. 8:**
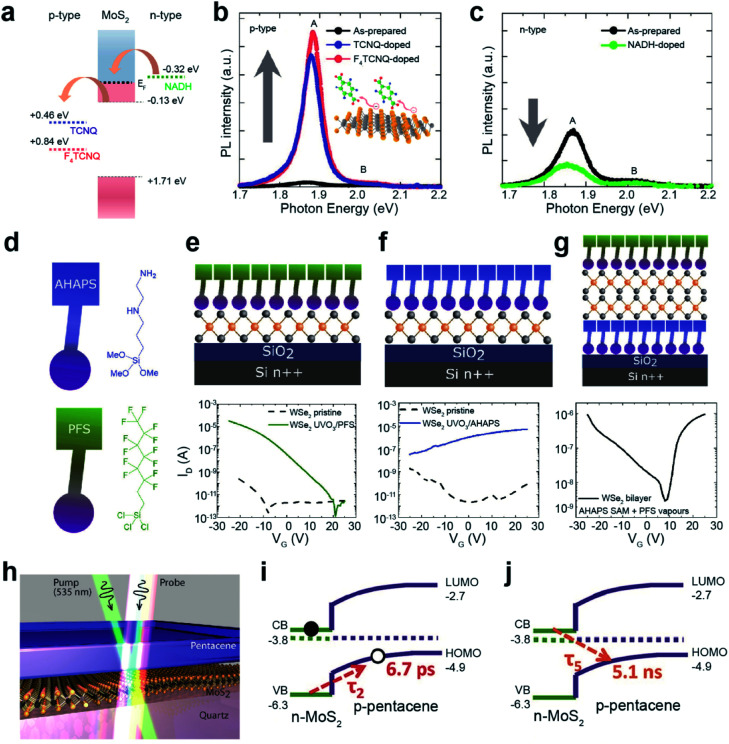
Noncovalent functionalization of 2D TMDs through chemical doping and heterojunction formation. **Chemical doping:** (a) reduction potentials (*vs.* SHE) of F_4_TCNQ and TCNQ for p-type doping and NADH for n-type doping. PL spectra of MoS_2_ after (b) p-type and (c) n-type doping. (d) Schematic of PFS and AHAPS. (e–g) Schematic of PFS-doped WSe_2_, AHAPS-doped WSe_2_, and double-side doped bilayer WSe_2_ with PFS and AHAPS (top) and their corresponding *I*–*V* transfer curves (bottom). **Heterojunction formation:** (h) illustration of the MoS_2_/pentacene p–n heterojunction probed by transient absorption spectroscopy. (i and j) Exciton dynamics of a type-II heterojunction. (a–c) Adapted with permission.^141^ Copyright 2013, American Chemical Society. (d–g) Adapted with permission.^142^ Copyright 2019, American Chemical Society. (h–j) Adapted with permission.^145^ Copyright 2017, American Chemical Society.

Chemical doping of organic molecules on carbon nanotubes (CNTs) and Gr has been extensively studied using two main approaches: (i) modulating the reduction potential of molecules (water bucket model)^137^ and (ii) controlling electron-donating (*e.g.*, amine, –NH_2_, and hydroxyl, –OH) and electron-withdrawing groups (*e.g.*, nitro, –NO_2_, and trihalogenated methyl, –CX_3_). Analogous strategies have been widely adopted for TMDs. The water bucket model describes the charge transfer between molecules and host materials induced by the difference in reduction potentials. Species with a lower reduction potential give electrons to those with a higher one.^137^ Material's reduction potential can be calculated by using the following equation: *φ*/*e* = *V* (V *vs.* SHE) + 4.44 V, where *φ*, *e*, *V*, and SHE denote the work function, electron, reduction potential, and standard hydrogen electrode, respectively.^138^ For MoS_2_, the reduction potentials of 0.84 eV (*vs.* SHE) for 2,3,5,6-tetrafluoro-7,7,8,8-tetracyanoquinodimethane (F_4_TCNQ) and 0.46 eV (*vs.* SHE) for 7,7,8,8-tetracyanoquinodimethane (TCNQ) indicate that they withdraw electrons from MoS_2_ (p-type doping), whereas −0.32 eV (*vs.* SHE) for nicotinamide adenine dinucleotide (NADH) indicates that they donate electrons (n-type doping) (Fig. 8a).^139,140^ A drastic enhancement in the PL intensity was observed for MoS_2_ after functionalization with F_4_TCNQ and TCNQ (Fig. 8b). In contrast, after functionalization with NADH an attenuation of the PL intensity was detected (Fig. 8c).^141^ These experimental results demonstrate the *E*_F_ shift of MoS_2_ according to p-type and n-type dopants.

Molecules with distinct functional groups can be n- or p-type dopants of TMDs. For example, mechanically exfoliated WSe_2_ flakes were exposed to vapors of trichloro(1*H*,1*H*,2*H*,2*H*-perfluorooctyl)silane (PFS) with –CF_3_ functional groups for p-type doping, and *N*-[3-(trimethoxysilyl)propyl]ethylenediamine (AHAPS) with –NH_2_ functional groups for n-type doping (Fig. 8d). Before this, however, WSe_2_ flakes were treated with ozone (UVO_3_) to improve the functionalization uniformity and ensure high surface coverage. The *I*–*V* transfer curves of the WSe_2_/PFS and WSe_2_/AHAPS FETs, fabricated on Si/SiO_2_ with Au contact electrodes, show p-type and n-type characteristics with enhanced hole and electron mobilities of 150 cm^2^ V^−1^ s^−1^ and 17.9 cm^2^ V^−1^ s^−1^, respectively (Fig. 8e and f). Moreover, asymmetric doping obtained by sandwiching bilayer WSe_2_ with AHAPS (bottom) and PFS (top) shows distinct ambipolar transport properties with electron and hole mobilities of 5.7 cm^2^ V^−1^ s^−1^ and 20 cm^2^ V^−1^ s^−1^, respectively (Fig. 8g).^142^ To achieve better ambipolar transport characteristics, monolayer WSe_2_ was subjected to a hybrid functionalization (covalent and non-covalent functionalizations) using two different dopants, 4-NBD and diethylenetriamine. It drastically enhanced the hole and electron mobilities to 82 cm^2^ V^−1^ s^−1^ and 25 cm^2^ V^−1^ s^−1^, respectively.^143^ In addition, a reduction in the energy bandgap (∼0.24 eV) was observed for WSe_2_, with asymmetric doping with C_60_F_48_ and graphite. This behavior is a consequence of the accumulation of holes and electrons at the bottom and top Se layers.^144^

Various heterojunctions can be easily constructed by the deposition of organic layers on TMD surfaces. Fig. 8h shows a typical prototype of a type-II organic/TMD heterojunction (pentacene/MoS). When an electron in MoS_2_ is excited by a photon, a hole is transferred to the p-type pentacene layer within a very short time (*τ*_2_ = 6.7 ps) (Fig. 8i). This enables the extension of the interlayer exciton lifetime to as long as ∼5 ns at the pentacene/MoS_2_ interface (Fig. 8j).^145^ This dynamic process originates from the quenching of the PL intensity of pentacene/MoS_2_ when compared to pristine MoS_2_. Similarly, MoS_2_/PTCDA, WS_2_/PTCDA, and MoS_2_/rubrene also exhibit a type-II p–n heterojunction.^134,146,147^ Such an extended charge recombination time can improve the quantum efficiency of optoelectronic devices such as diodes, bipolar transistors, photodiodes, and so on.^148–150^

#### Applications

**Fig. 9:**
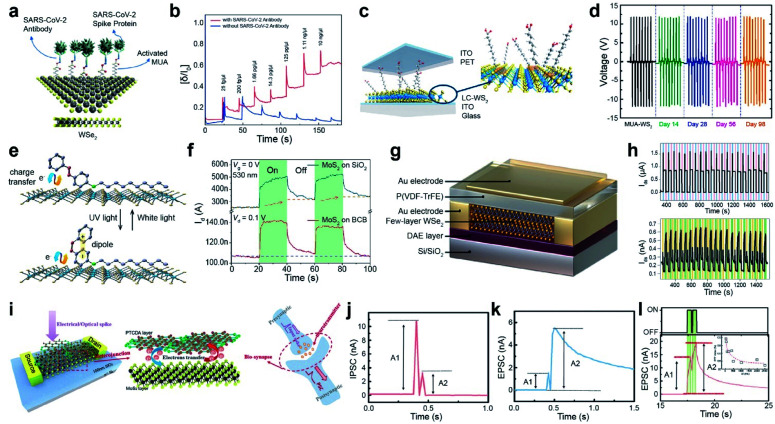
Functionalization for various advanced applications: CoVID-19 sensors, TENGs, multifunctional optoelectronic device, and memory and neuromorphic devices. **CoVID-19 sensors:** (a) schematic of 11-MUA-functionalized WSe_2_ for a CoVID-19 sensor. (b) Real-time detection of various SARS-CoV-2 antigen spike proteins with (red curve) and without antibodies (blue curve) using *V*_DS_ = 1 V and *V*_GS_ = −0.5 V with a functionalized WSe_2_ FET. **TENGs:** (c) Schematic of a ligand-conjugated WS_2_ TENG device. (d) Output voltages of pristine WS_2_ and ligand-conjugated WS_2_ TENG devices. **Multifunctional optoelectronic device:** (e) schematic of a photomolecular switch device with photochromic molecules (4-(decyloxy)azobenzene) on MoS_2_. (f) Time-resolved photoresponse of MoS_2_ FETs on BCB and SiO_2_ substrates under 530 nm light illumination. **Memory device:** (g) schematic of the DAE/WSe_2_/FeFET device. (h) Switching behavior for 20 cycles induced by applying a cyclic ±60 V pulse bias (top) and UV-Vis illumination (bottom). **Neuromorphic device:** (i) schematic of a MoS_2_/PTCDA heterojunction operated under both electrical/optical pulses (presynaptic input) and corresponding output current (postsynaptic output). The electron transfer process at the heterojunction interface is analogous to the release of neurotransmitters in biosynapses. (j and k) IPSC/EPSC behavior triggered by a pair of relatively negative *V*_cg_ (gate voltage) pulses. (l) EPSC behavior stimulated by a pair of laser pulses. The inset shows the corresponding PPF index *A*_2_/*A*_1_ as a function of laser pulse interval time. (a and b) Adapted with permission.^154^ Copyright 2021, American Chemical Society. (c and d) Adapted with permission.^158^ Copyright 2021, American Chemical Society. (e and f) Adapted with permission.^160^ Copyright 2019, American Chemical Society. (g and h) Adapted with permission.^162^ Copyright 2021, Wiley-VCH. (i–l) Adapted with permission.^163^ Copyright 2019, Wiley-VCH.

COVID-19 sensors, TENGs, multifunctional optoelectronic devices, and memory and neuromorphic devices are some of the applications discussed here. The development of rapid diagnostic tools for the detection of severe acute respiratory syndrome coronavirus 2 (SARS-CoV-2) is highly needed. Real-time reverse transcription-polymerase chain reaction (RT-PCR) is the only specific diagnostic test based on unique sequences of viral ribonucleic acid (RNA). It is, however, very time-consuming. Hence, having rapid, effective, label-free, point-of-care, and low-cost sensors for detecting viral antigens is crucial.^151–153^ WSe_2_ monolayers were functionalized with 11-mercaptoundecanoic acid (MUA) and activated with *N*-hydroxysuccinimide (NHS) for the detection of SARS-CoV-2 spike proteins (Fig. 9a). Fig. 9b illustrates the response of the device during real-time detection of SARS-CoV-2 under different concentrations of the spike protein. The number of antibodies with SARS-CoV-2 spike protein increased with the concentration (red line), while antibodies without SARS-CoV-2 spike protein showed a slight decrease (blue line). Moreover, the biosensor based on WSe_2_ FETs after functionalization with MUA can detect SARS-CoV-2 spike protein down to 25 fg μL^−1^ in 0.01 M phosphate-buffered saline (PBS) solution,^154^ which is ∼10^4^ orders of magnitude higher than the detectivity of Gr-based biosensors (1 fg mL^−1^).^155^

With the rapid development of the Internet of Things (IoT), over 50 billion IoT sensors already exist and are expected to surpass 200 billion by 2025.^156,157^ Among various energy harvesters, TENGs can convert waste mechanical energy into electrical energy under ambient conditions. WS_2_ nanosheets were functionalized *via* thiol conjugation reactions at S_V_ sites with various alkanethiol molecules, such as mercaptopropionic acid (MPA), mercaptohexanoic acid (MHA), mercaptooctanoic acid (MOA), and MUA. A TENG device was fabricated using functionalized WS_2_ and poly(ethylene terephthalate) (PET) as the negative and positive active layers, respectively. Indium tin oxide was used as an electrode (Fig. 9c). Thiol-containing ligands with different alkane chain lengths act as triboelectrification layers in TENGs. A high-precision micromechanical tester was used to press and release a TENG device with a vertical force of 6 N and a frequency of 1 Hz. Pristine WS_2_ and alkanethiol-functionalized WS_2_ TENGs exhibited an output voltage of 1.1 V, 8.8 V (MPA), 10.6 V (MHA), 11.4 V (MOA), and 12.2 V (MUA) under open-circuit conditions (Fig. 9d). The MUA-WS_2_ TENG exhibited a maximum power density of 138 mW m^−2^. The persistent output voltage of the MUA-WS_2_ TENG device was maintained after 14, 28, 56, and 98 days, confirming high stability and durability.^158^ This indicates that alkanethiol functionalization of defective WS_2_ surfaces *via* ligand conjugation suppresses reactions with reactive oxygen and reduces the number of catalytically active sites. Moreover, a stable triboelectric output voltage was observed for the MUA-WS_2_ TENG device even after 10 000 cycles. Several 2D materials, including MoS_2_, WS_2_, MoSe_2_, Gr, and Gr oxide (GO)-based TENGs, were fabricated to utilize their triboelectric charging nature. The work functions of the materials decreased in the following order: MoS_2_ (4.85 eV) > MoSe_2_ (4.70 eV) > Gr (4.65 eV) > GO (4.56 eV) > WS_2_ (4.54 eV). Hence, MoS_2_ is likely to be triboelectrically more negative than the other materials. Furthermore, MoS_2_ is functionalized with benzyl viologen (BV, n-type) and gold chloride (AuCl_3_, p-type), resulting in positive and negative values over a triboelectric series (by changing the work function accordingly). These results suggest that triboelectric charging can be tuned through functionalization.^159^

Another example of the application is a photomolecular switch device, which was fabricated using thin photochromic azobenzene (AZO) physisorbed on MoS_2_ that was a trap-free benzocyclobutene (BCB)/SiO_2_/Si substrate (Fig. 9e). The AZO molecules on MoS_2_ undergo reversible isomerization between the *cis* and *trans* states upon exposure to white and UV light illumination, which efficiently modulates the charge transfer process between AZO and the underlying 2D MoS_2_. The photoresponse of the MoS_2_ FET device on BCB represents a fast saturation under 530 nm light illumination for 20 s, whereas it shows a steady increase for MoS_2_ on a SiO_2_/Si substrate (Fig. 9f). This high response originates from the suppression of the persistent photoconductivity (PPC) effect. Furthermore, a high thermal stability of over 15 h was demonstrated for the photomolecular switching of metastable *cis/trans* states.^160^ In addition, a type-II heterojunction phototransistor was developed using an organic phosphonic acid monolayer (12-(benzo[*b*]benzo[4,5]thieno[2,3-*d*]thiophen-2-yl)dodecyl) (BTBT) stacked over a MoS_2_ monolayer. It exhibits an unprecedented responsivity of 475 A W^−1^ and high external quantum efficiency of 1.45 × 10^5^%. BTBT provides effective charge transfer *via* π–π interactions and successfully eliminates recombination and charge scattering.^161^

A ternary-responsive multilevel memory device was fabricated with few-layer WSe_2_*via* asymmetric non-covalent functionalization. A photochromic diarylethene (DAE) layer functionalized WSe_2_ at the bottom, while a ferroelectric poly(vinylidene fluoride-trifluoroethylene) (P(VDF-TrFE)) layer was placed on the top of WSe_2_ to form a multi-stimuli-responsive Janus WSe_2_ FET device (Fig. 9g). The device successfully generated two states because of the polarization of P(VDF-TrFE) in the downward/upward direction for a cyclic ±60 V pulse bias. One cycle consisted of +60 V for 2 s (blue region), 0 V for 30 s, −60 V for 2 s (violet region) and 0 V for 30 s (Fig. 9h (top)). Switching with a photochromic DAE was also demonstrated under UV-Vis illumination, where one cycle consisted of 5 s under UV illumination (orange region), 20 s in the dark, 20 s under Vis light (green region), and 20 s without illumination (Fig. 9h (bottom)). Moreover, a multistimuli-responsive asymmetrically functionalized Janus WSe_2_ device was successfully achieved by modulating the population ratio of polarized P(VDF-TrFE) and photoisomerization DAE. This generated nine unique ferroelectric states and 84 photogenerated states, respectively. The overall maximum of 756 current levels was stored in a single device. The cyclic endurance (10 cycles) and data retention (over 1000 h) confirm the consistency of the device and present high-density non-volatile memory devices.^162^

A multifunctional neuromorphic device was fabricated *via* non-covalent functionalization of perylene-3,4,9,10-tetracarboxylic dianhydride (PTCDA) on MoS_2_, which forms a type-II heterostructure (Fig. 9i). The charge transfer phenomenon between MoS_2_ and PTCDA mimics neurotransmitter release in biological synapses (right panel of Fig. 9i). The MoS_2_/PTCDA hybrid synaptic transistor was operated under an electrical field by applying a pair of negative gate voltage (*V*_cg_) pulses (−12 V for 25 ms and −20 V for 25 ms). It exhibited inhibitory post-synaptic current (IPSC) behavior (*A*_2_ < *A*_1_) and excitatory post-synaptic current (EPSC) behavior (*A*_2_ > *A*_1_), which correspond to synaptic paired-pulse depression (PPD) and paired-pulse facilitation (PPF) behavior, respectively (Fig. 9j and k). Similarly, a typical EPSC behavior is observed by applying a pair of 532 nm laser pulses (green irradiation with a pulse width of 400 ms, an interval of 100 ms, and a *V*_DS_ of 0.1 V) (Fig. 9l). Under both operating modes, the type-II MoS_2_/PTCDA hybrid heterojunction synaptic device performed consistently and successfully mimicked biological synapse functions by an efficient charge transfer process at the hybrid interface.^163^

### 2.4. Repair

Atomic chalcogen vacancies in TMDs attenuate their electrical and optical properties (as seen by low PLQY, high Schottky barriers, and limited carrier mobility), which is translated to poor electronic and optoelectronic device performances.^164–169^ Therefore, the repair of atomic defects is critical for the realization of high-performance devices. Atomic chalcogen vacancies can be repaired using the same chalcogens for healing and other elements for passivation.

#### Healing

**Fig. 10:**
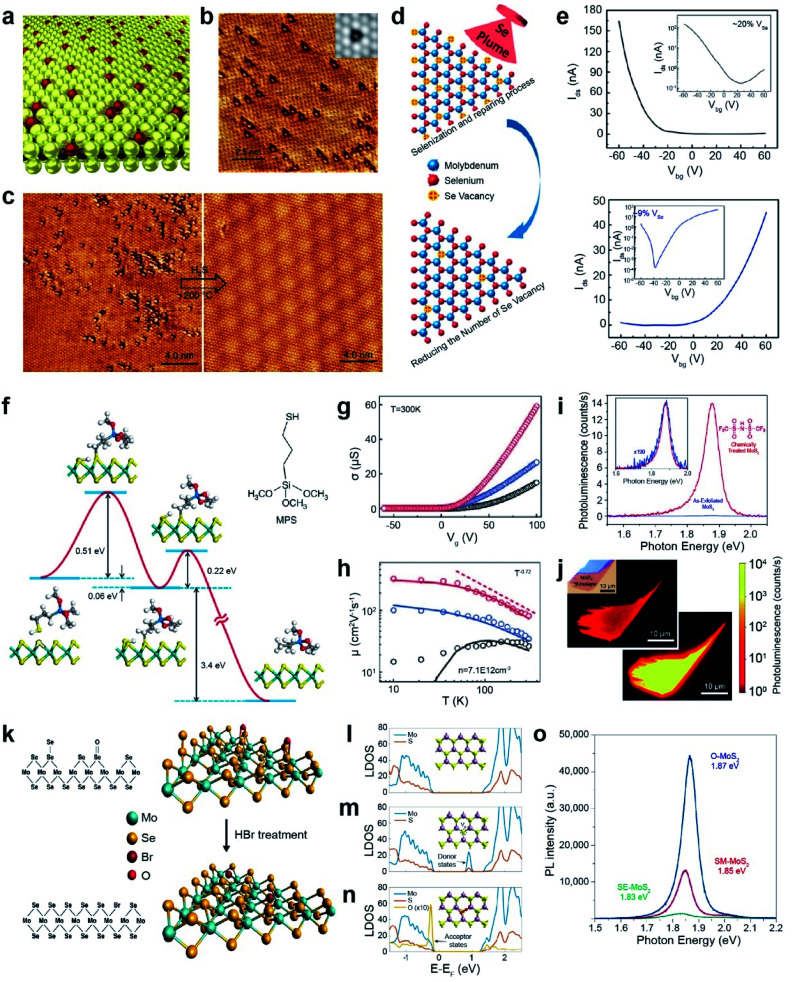
Repair of 2D TMDs: healing and passivation. **Healing:** (a) schematic representation of atmospheric oxygen atoms getting adsorbed on a MoS_2_ monolayer. (b) STM image of O atoms (bright spots) absorbed at S_V_ (dark triangles) sites. Inset: simulated STM image. (c) STM images of MoS_2_ before and after H_2_S healing at 200 °C. (d) Schematic illustration for post-selenization repair of Se_V_ in MoSe_2_. (e) Transfer curves of high Se_V_ (∼20%) (top) and healed Se_V_ (∼9%) (bottom) samples. (f) Illustration of reaction pathways for repairing S_V_-MoS_2_ using 3-MPS organic molecules. (g and h) Electrical conductance (*σ*) as a function of gate voltage, carrier mobility and measurement temperature of exfoliated (black), single side treated (blue) and double side treated (red) samples. (i) PL spectra of exfoliated MoS_2_ (blue) and TFSI-treated MoS_2_ (red). The inset shows the 190× magnified PL spectra of as-exfoliated MoS_2_. (j) Confocal PL mapping image of exfoliated MoS_2_ (top) and TFSI-treated MoS_2_ (bottom). **Passivation:** (k) schematic of Se_V_ passivation with Br in MoSe_2_ by HBr treatment and the corresponding atomic structures. (l–n) LDOS of (l) pristine MoS_2_ (top), (m) S_V_-MoS_2_ (middle), and (n) O-MoS_2_ (bottom). (o) PL spectra of SE-MoS_2_, SM-MoS_2_, and O-MoS_2_ grown under oxygen conditions (O-MoS_2_), sulfur-mild conditions (SM-MoS_2_) and sulfur-excess conditions (SE-MoS_2_), respectively. (a–c) Adapted with permission.^173^ Copyright 2018, Springer Nature Limited. (d and e) Adapted with permission.^174^ Copyright 2016, American Chemical Society. (f–h) Adapted with permission.^177^ Copyright 2014, Springer Nature Limited. (i and j) Adapted with permission.^180^ Copyright 2015, American Association for the Advancement of Science. (k) Adapted with permission.^182^ Copyright 2016, American Chemical Society. (l–o) Adapted with permission.^186^ Copyright 2022, Springer Nature Limited.

Hydrosulfurization and post-sulfuration/selenization treatments effectively heal chalcogen vacancies in TMDs.^170^ For example, atmospheric oxygen atoms can easily react with S_V_ or Se_V_ and passivate CVD-grown or exfoliated TMDs (Fig. 10a).^171,172^ Two possible reactions can occur between oxygen atoms and TMDs: (i) formation of oxygen-substituted TMDs (MoS_2−*x*_O_*x*_) and (ii) creation of SO_2_ or SeO_2_ volatile compounds. DFT calculations show that the removal of S atoms is thermodynamically favorable (*E* = −0.49 eV, negative oxidation enthalpy), whereas it is unfavorable for Se atoms (*E* = +0.75 eV, positive oxidation enthalpy). Bright triangular spots corresponding to single O atoms were observed in the scanning tunneling microscope (STM) image (Fig. 10b). Such MoS_2−*x*_O_*x*_ compounds are completely healed and transformed to defect-free MoS_2_ by simple thermal annealing at 200 °C under a H_2_S atmosphere (Fig. 10c).^173^ It is noted that MoS_2−*x*_O_*x*_ shows better HER performance than defect-free MoS_2_ because the presence of oxygen atoms in TMDs decreases the Δ*G*_H_ for hydrogen adsorption, by bringing it closer to thermoneutral conditions.^173^

The post-selenization process is performed on defected monolayer MoSe_2_*via* pulsed laser vaporization of selenium to repair Se_V_ (Fig. 10d). The temperature plays a crucial role in selenization, where the optimum temperature range is between 600 and 700 °C. The *I*–*V* transfer curves show major carrier type conversion from p-type characteristics for high Se vacancies (∼20% V_Se_) to n-type characteristics for healed Se vacancies (∼9% V_Se_). Moreover, the obtained hole and electron mobilities were ∼0.011 and 0.021 cm^2^ V^−1^ s^−1^, respectively (Fig. 10e).^174^ Selective post-sulfuration on patterned MoSe_2_ readily forms MoSe_2_/MoS_2_ heterojunctions and exhibits a type-I band alignment.^175^

Another healing method for S_V_ in MoS_2_ (S_V_-MoS_2_) involves the use of S-containing organic molecules.^176^ For example, S_V_-MoS_2_ was coated with 3-mercaptopropyl trimethoxysilane (MPS) and subsequently annealed at 350 °C under a H_2_/Ar atmosphere. The healing process involves the following reaction: HS(CH_2_)_3_Si(OCH_3_)_3_ + S_V_-MoS_2_ → CH_3_(CH_2_)_2_Si(OCH_3_)_3_ + MoS_2_. The reaction kinetics of S_V_-MoS_2_ and MPS involve two representative steps of chemical adsorption between S_V_ and thiol groups in MPS and dissociation of the S–C bond with an energy barrier of 0.51 and 0.22 eV, respectively (Fig. 10f). The S_V_ density was dramatically reduced from ∼6.5 × 10^13^ to ∼1.6 × 10^13^ cm^−2^, for topside MPS treatment. Electrical transport properties were measured for three FET samples on Si/SiO_2_: exfoliated MoS_2_ (black), top-side-treated MoS_2_ (blue), and double-side-treated MoS_2_ (red) (Fig. 10g). The double-sided MPS treatment further reduced short-range scattering and charge impurities and thus enhanced the carrier mobility of 81 cm^2^ V^−1^ s^−1^ at room temperature.^177^ The carrier mobility of these samples at low temperature (10 K) was further increased to 14 cm^2^ V^−1^ s^−1^, 106 cm^2^ V^−1^ s^−1^, and 320 cm^2^ V^−1^ s^−1^, respectively (Fig. 10h). The catalytic properties of MPS-treated MoS_2_ drastically decrease due to the depletion of electrochemically active sites, thereby increasing the overpotential and the Tafel slope.^178^

As an alternative, bis(trifluoromethane) sulfonamide (TFSI) was employed to heal S_V_ in MoS_2_ and WS_2_.^179^ The exfoliated MoS_2_ that was treated with TFSI exhibited a 190-fold increase in the magnitude of the PL peak intensity (Fig. 10i), consisting of a brighter PL image than that of the pristine one (Fig. 10j).^180^ An increased quantum yield QY (>95%) and longer lifetime (∼10 ns) were also observed due to the elimination of the non-radiative recombination. TFSI-treated MoSe_2_ and WSe_2_ exhibited a moderately reduced QY. TEM analysis revealed that Se_V_ sites in WSe_2_ were not passivated by S atoms.^181^

#### Passivation

Passivation has been conducted not only to improve the optical and electrical properties of TMDs but also to stabilize their structures. To enhance the PL intensity of MoSe_2_, the passivation of Se with Br atoms was introduced (Fig. 10k).^182^ CVD-grown MoSe_2_ shows a 30-fold increase in PL intensity after HBr treatment due to Se_V_ passivation and p-doping effects.^182^ For example, the electrical and optical properties of WSe_2_ and ReSe_2_ can be improved *via* HCl treatment. The halogen atoms efficiently repair the Se_V_ sites and shift the defective states from the donor level to the acceptor level (*i.e.* transforming from n-type to p-type).^183–185^ Single S_V_ or point defects can be efficiently passivated by oxygen atoms. The local density of states (LDOS) of pristine MoS_2_, S_V_-MoS_2_, and O-MoS_2_ shows the annihilation of the donor state in S_V_-MoS_2_ and the creation of an acceptor state below the top of the valence band in O-MoS_2_ (Fig. 10l–n).^186^ Three different conditions were adopted for the preparation of three distinct MoS_2_ monolayers by growing them under oxygen (O-MoS_2_), sulfur-mild (SM-MoS_2_), and sulfur-excess (SE-MoS_2_) conditions. The neutral A-exciton peak intensity of the O-MoS_2_ is dominant in the PL spectra(Fig. 10o). This is ascribed to suppressing the non-radiative recombination and p-doping effect by new acceptor states.^186^

The healing of atomic chalcogen vacancies using organic thiol molecules is still under debate. Three possible reaction mechanisms include repair, functionalization, and dimerization.^187^ The energy barrier rate-determining steps for both functionalization and repair mechanisms are almost similar, indicating that these are competing reactions.^188^ Moreover, numerous factors, such as the nature of thiol molecules, concentration of S_V_, reaction temperature, and time, are critical in determining the reaction mechanism. Furthermore, thiophenol molecules (C_6_H_5_SH) can heal and adsorb on Se_V_ in WSe_2_. The adsorbed thiophenol molecule displays a vertical configuration, which is consistent with experimental STM images.^189^ The mechanisms of defect passivation and/or healing are still not clearly understood for TFSI-treated TMDs. TFSI treatment increases the PL lifetime but limits the carrier mobility by the charge scattering mechanism.^181,190^ In order to overcome this issue, exfoliated MoS_2_ and WS_2_ were first treated with thiol molecules (3-*n*-octylthiophene, dipropyl sulfide, or ethanethiol) to control S_V_, followed by TFSI treatment. The two-step treatment effectively eliminates sub-gap states and lowers the Fermi level, which greatly improves the PL lifetime and enhances carrier mobility.^191^

#### Application

**Fig. 11:**
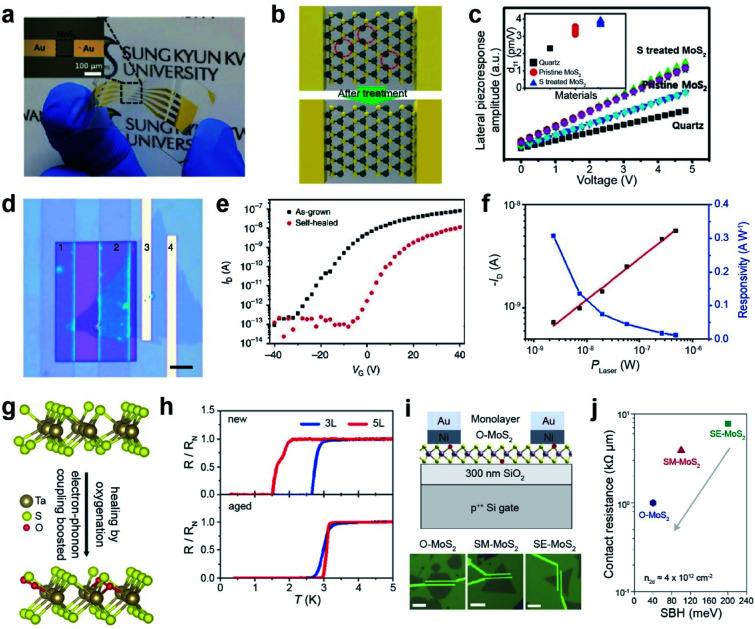
Repair-based various applications: PENG, photodiode, superconductors, and contact resistance in FETs. **PENG:** (a) photograph of a flexible PENG device on a PET substrate. (b) Schematic of S_V_ healing in MoS_2_*via* H_2_S treatment. (c) Lateral piezoelectric effect of quartz, S_V_-MoS_2_, and S_V_-treated MoS_2_ samples. **Photodiode:** (d) optical image of (1–2) a selectively self-healed (PEDOT:PSS) MoS_2_ FET, (2–3) a self-healed MoS_2_/as-grown MoS_2_ homojunction, and (3–4) as-grown MoS_2_ FET devices (scale bar: 5 μm). (e) *I*–*V* transfer curve of as-grown MoS_2_ (black) and self-healed MoS_2_ (red). (f) Photocurrent and responsivity on the (2–3) MoS_2_ homojunction device. **Superconductors:** (g) schematic of oxygen passivated TaS_2_. (h) Normalized resistance as a function of temperature for new and aged (after 3 weeks) devices. **Contact resistance in FETs:** (i) schematic of the as-fabricated oxygen-rich MoS_2_ (O-MoS_2_) FET on SiO_2_/Si and the corresponding optical images of O-MoS_2_, SM-MoS_2_ and SE-MoS_2_ with a 1 μm channel length. (j) Contact resistance (*R*_C_) *versus* Schottky barrier height (SBH) for the three devices. (a–c) Adapted with permission.^193^ Copyright 2018, Wiley-VCH. (d–f) Adapted with permission under a Creative Commons CC-BY License.^194^ Copyright 2017, Springer Nature Limited. (g and h) Adapted with permission.^198^ Copyright 2020, American Chemical Society. (i and j) Adapted with permission.^186^ Copyright 2022, Springer Nature Limited.

Successfully repaired TMDs can be utilized in various applications, including flexible PENGs, superconductors, FET contact resistance, and photodiodes. Monolayer 2D TMDs exhibit strong piezoelectric properties when an external force is applied because of their broken inversion symmetry.^192^ A flexible PENG was fabricated by using healed MoS_2_, obtained *via* a thermal annealing treatment at 1000 °C for 30 min under H_2_S gas (Fig. 11a and b). The lateral piezoelectric response was measured by applying an external electric field between two electrodes. Fig. 11c illustrates the piezoelectric responses of as-grown monolayer MoS_2_, S-treated MoS_2_ (healed MoS_2_), and α-quartz as a function of the applied external bias. The piezoelectric output current and voltage were measured under a tensile strain of 0.48% with a strain rate of 70 mm s^−1^. The piezoelectric response of the S-treated MoS_2_ surpasses that of the pristine monolayer MoS_2_ and α-quartz. The inset in Fig. 11c shows that the piezoelectric coefficients (*d*_11_) are 3.73 pm V^−1^, 3.06 pm V^−1^, and 2.3 pm V^−1^ for S-treated MoS_2_, pristine MoS_2_, and α-quartz, respectively. The output current and voltage generated by the S-treated MoS_2_ PENG device (100 pA and 22 mV) are ∼3 and 2 times higher than those of the pristine MoS_2_ PENG device (30 pA and 10 mV), respectively.^193^

A photodiode was fabricated using selective healing of patterned MoS_2_. Self-healing is carried out by the hydrogenation of poly(3,4-ethylenedioxythiophene)–poly(4-styrenesulfonate) (PEDOT:PSS). Fig. 11d displays the optical image of the selectively healed MoS_2_ FETs, where regions 1 and 2 are healed MoS_2_, regions 3 and 4 are as-grown MoS_2_ FETs, and regions 2 and 3 indicate homojunctions. The transfer characteristics of the as-grown MoS_2_ and self-healed MoS_2_ are shown in Fig. 11e. *V*_T_ shifted toward zero after self-healing, indicating unipolar n-type electrical transport behavior. The MoS_2_ homojunction photodiode exhibits a high photoresponsivity of 308 mA W^−1^ (observed at zero source-drain bias) with excellent air stability (Fig. 11f), as a result of efficient electron–hole separation at the homojunction.^194^

Recently, research on ultrathin superconductors has been exclusively conducted after the advancement in exfoliation (from bulk down to monolayer) and encapsulation techniques of 2D materials. However, monolayer TMDs possess a high density of defects, eventually resulting in the localization of Cooper pairs and decreasing transition temperature (*T*_c_) for metal-to-insulator transitions.^195–197^ S_V_ sites in ultrathin TaS_2_ are passivated by oxygen to form oxygenated TaS_2_ in air (Fig. 11g). Theoretical calculations predict that oxygen passivation in monolayer TaS_2_ significantly decreases the carrier density of pure TaS_2_. Transport measurements were performed for three- (3L) and five-layered (5L) TaS_2_ devices, either in a fresh (denoted by new) or oxygenated (denoted by aged) form (Fig. 11h). The *T*_c_ values of both 3L and 5L TaS_2_ increase with oxygen, owing to the increase in electron–phonon coupling.^198^

FETs were fabricated using three different types of monolayer MoS_2_: those grown under oxygen conditions (O-MoS_2_), mild sulfur conditions (SM-MoS_2_), and excess sulfur conditions (SE-MoS_2_) (Fig. 11i). The *I*–*V* transfer curves of O-MoS_2_ display a positive *V*_th_ (+21.0 ± 4.6 V), whereas a negative *V*_th_ was observed for SE-MoS_2_ (−17.0 ± 9.7 V) and SM-MoS_2_ (2.8 ± 4.9 V). The contact resistance (*R*_C_) of the three devices was determined using the Schottky barrier height (SBH) at the interface (Fig. 11j).^186^ The O-MoS_2_ FET exhibits a low *R*_C_ value of ∼1 kΩ μm, whereas high *R*_C_ values of 3.9 and 7.8 kΩ μm are observed for SM-MoS_2_ and SE-MoS_2_ FETs, respectively (at the same carrier density (*n*_2D_) of 4 × 10^12^ cm^−2^). This is ascribed to the reduced SBH in O-MoS_2_ owing to the absence of donor states and the Fermi level closer to the CBM of MoS_2_.

## 3. Structural modification

This section presents a discussion on two structural modifications of TMDs for various device applications: (i) phase transition and (ii) heterostructure formation. In the first sub-section, the structural phase transition from trigonal prismatic 2H to octahedral 1T or distorted 1T′ is addressed, with potential applications in wireless energy harvesting, solar cells, electrocatalysts, Li-ion batteries, and memory and neuromorphic devices. In the second sub-section, the construction of vertical van der Waals and lateral heterostructures is described, with several applications, such as photocatalysts, photodetectors, solar cells, magnetic applications, and sensors.

### 3.1. Phase transition

TMDs exist in a wide range of crystalline phases, from trigonal prismatic 2H to octahedral 1T or distorted 1T.^199^ The stability and the free energy between the phases differ, depending on the material. For example, 2H-MoS_2_ is more stable than 1T′-MoS_2_ under ambient conditions and the free energy difference between the two phases is very large (Δ*E* > 0.8 eV). In contrast, MoTe_2_ exhibits a small free energy difference (Δ*E* < 50 meV) between the 2H and 1T′ phases.^200,201^ Therefore, techniques for phase transition are unique for each material. Metallic phase accelerates the electron transport to obtain low contact resistance of FETs and enhances their performance in electrocatalysis, supercapacitors, and batteries.

**Fig. 12:**
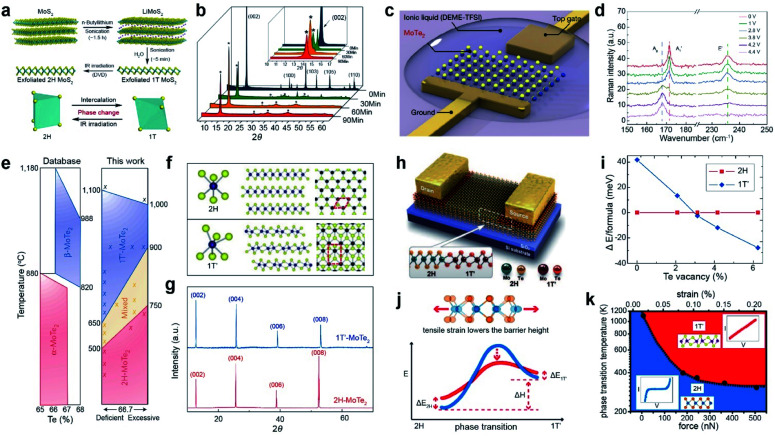
Phase transition of 2D TMDs with Li intercalation, electrostatic doping, thermal annealing, laser, and tensile strain. **Li intercalation:** (a) schematic illustration of Li intercalation into MoS_2_. (b) XRD patterns obtained during the sonication-assisted lithium intercalation of MoS_2_ for different times. **Electrostatic doping:** (c) schematic of a monolayer MoTe_2_ FET. (d) Raman spectra of phase transition from the 2H to the 1T′ phase with bias changed from 0 V to 4.4 V. **Thermal annealing:** (e) phase diagram of MoTe_2_ from the ASM alloy phase diagram database (left) and the new phase diagram based on experiment (right). (f) Ball-and-stick models for 2H- and 1T′-MoTe_2_. (g) XRD patterns of 1T′-MoTe_2_ (blue) and 2H-MoTe_2_ (red) single crystals. **Laser treatment:** (h) schematic of a 1T′/2H MoTe_2_ homojunction device. (i) DFT-calculated energy difference between the 2H and 1T′ phases as a function of Te_V_ concentration. **Tensile strain:** (j) schematic of the strain-dependent phase transition barrier. (k) Temperature- and force-dependent phase diagram of semiconducting 2H and metallic 1T′ MoTe_2_. (a and b) Adapted with permission.^203^ Copyright 2011, American Chemical Society. (c and d) Adapted with permission.^210^ Copyright 2017, Springer Nature Limited. (e–g) Adapted with permission.^216^ Copyright 2015, Springer Nature Limited. (h and i) Adapted with permission.^222^ Copyright 2015, American Association for the Advancement of Science. (j and k) Adapted with permission.^224^ Copyright 2016, American Chemical Society.

Li intercalation effectively reduces the energy barrier of the phase transition from 2H-MoS_2_ to 1T-MoS_2_ and stabilizes the metallic phase.^202^*n*-Butyllithium in hexane was employed for Li intercalation in MoS_2_ and it induced a phase transition from 2H to 1T. This was followed by sonication of LiMoS_2_ to exfoliate 1T MoS_2_ (∼1–3 nm film) in water (Fig. 12a).^203^ The 1T-MoS_2_ thin films, prepared by drop-casting on a DVD disc, were reverted to the 2H phase by IR laser irradiation, as confirmed by XRD (Fig. 12b). The characteristic (002) peaks for 2H-MoS_2_ disappeared after 60 min, and new diffraction peaks (indicated by stars) appeared, which correspond to the 1T phase. Furthermore, electrochemical lithiation has been proposed to replace the flammable and dangerous *n*-butyllithium. Li foil and MoS_2_ were used as anodes and cathodes, respectively.^204,205^ Alternatively, alkali metals (lithium, potassium, or sodium) and naphthalene can be used for phase transitions.^206–209^

A low free energy difference (<50 meV) between the 2H and 1T′ phases in MoTe_2_ enables a reversible phase transition. For example, electrostatic doping with an ionic liquid (*N*,*N*-diethyl-*N*-(2-methoxyethyl)-*N*-methylammoniumbis(trifluoromethyl sulphonyl-imide) and DEME-TFSI) gating in a FET triggers a reversible phase transition (Fig. 12c).^210,211^ The Raman spectra of MoTe_2_ exhibit the gradual disappearance of the A′_1_ mode (171.5 cm^−1^) and E′ mode (236 cm^−1^) of 2H MoTe_2_ with increasing gate voltage from 0 V to 4.4 V, in addition to the appearance of the A_g_ mode (165.5 cm^−1^) of 1T′ MoTe_2_ (Fig. 12d). A fully reversible phase transition of MoTe_2_ can be achieved by increasing or decreasing the gate voltage. Similarly, the electrochemical phase transition of MoTe_2_ (from a monolayer to a 73 nm thick sample) was achieved using ionic liquid gating at room temperature in air.^212^ The phase transition of T-TaS_2_ (from T to H) and NbSe_2_ (from 2H to 1T) was achieved by applying a high bias voltage under a scanning tunneling microscope (STM) tip at low temperatures.^213,214^ The high density of electron doping from the 2D electrode [Ca_2_N]^+^·e^−^ drives the phase transition from the 2H to the 1T′ phase in a long MoTe_2_ (up to ∼100 nm) sample.^215^

In addition, the phase diagram of MoTe_2_ indicates that the phase stability of 2H and 1T′ strongly depends on temperature and Te deficiency (Fig. 12e). Only the 1T′ phase was obtained by rapid cooling at 900 °C during the flux synthesis process, whereas the 2H phase was obtained by slow cooling to room temperature, as confirmed by XRD patterns (Fig. 12f and g).^216^ On the other hand, a phase transition from 1T′ to 2H MoTe_2_ has been demonstrated with thermal annealing at 650 °C under a Te rich atmosphere.^217^ Recently, single-crystal multilayer 2H MoTe_2_ films were successfully synthesized *via* a phase transition from a polycrystalline multilayer 1T′ MoTe_2_ film initiated from single-crystal 2H MoTe_2_ seeds.^218^ The phase transitions of other TMDs, such as TaS_2_, PtSe_2_, and PdSe_2_, were also demonstrated with thermal annealing processes.^219–221^

A homojunction between the 2H and 1T′ phases in the MoTe_2_ FET was realized by a laser-driven phase transition (Fig. 12h). The metallic 1T′ phase was induced by selective laser irradiation of the 2H region to introduce Te vacancies (Fig. 12i). This homojunction in MoTe_2_ FET devices significantly reduces the contact resistance by forming an ohmic contact between the 1T′ and 2H regions.^222^ Similarly, the metastable 1T/1T phase of MoS_2_ is changed to a stable 2H phase *via* continuous-wave laser and femtosecond pulsed laser radiation.^223^

The introduction of strain is another strategy for inducing a phase transition. The tensile strain lowered the barrier height for the phase transition from 2H-MoTe_2_ to 1T′-MoTe_2_ (Fig. 12j). The temperature is further required to overcome the energy barrier for the phase transition under tensile strain (Fig. 12k). Experimentally, the reversible phase transition between 2H and 1T′ of MoTe_2_ was confirmed using an atomic force microscope (AFM) tip.^224^ Supercritical CO_2_ treatment can also induce a phase transition in TMDs *via* the formation of local strain in MoS_2_.^225,226^ A chalcogen vacancy is also used to induce local strain, resulting in the spatial phase transition of MoS_2_ and PdSe_2_.^227,228^

Phase transitions in TMDs can be achieved by alkali metal intercalation, electrostatic doping, electron transfer, thermal treatment, external irradiation, and strain. A summary of the phase transitions of the TMDs is listed in Table 2. Reversible phase transition between 2H and 1T (1T′) can be induced by Li intercalation/annealing as well as ionic liquid gating. However, Li intercalation, plasma treatment, and supercritical CO_2_ can't reach 100% yield of metallic phase TMDs.

**Table 2:** Summary of representative phase transition methods of TMDs

Materials	Phase	Methods	Application	References
MoS_2_	2H → 1T	Li intercalation	—	203
MoS_2_	2H → 1T	Na and naphthalenide intercalation	—	206
MoS_2_	2H → 1T′	Li intercalation	—	207
MoS_2_	2H → 1T	Electrochemical Li	HER	208
MoS_2_	2H → 1T/1T′	K intercalation	—	209
MoTe_2_	2H → 1T′	Electrostatic doping	—	211
NbSe_2_	2H → 1T	Electrostatic doping	—	214
MoTe_2_	2H → 1T′	Electron transfer ([Ca_2_N]^+^·e^−^)	—	215
MoTe_2_	2H → 1T′	Thermal treatment	—	216
TaS_2_	1T → 2H	Thermal treatment	—	219
PtSe_2_	1T ↔ 1H	Thermal treatment	—	220
MoTe_2_	2H → 1T′	Laser irradiation	FET	222
MoS_2_	1T/1T′ → 2H	Laser irradiation	—	223
MoTe_2_	2H → 1T′	Tensile strain (AFM tip)	—	224
MoS_2_	2H → 1T′	Supercritical CO_2_	HER	225
MoS_2_	2H → 1T	Ar plasma	FET	227
MoS_2*x*_Se_2(1−*x*)_	2H → 1T	Electrochemical Li	Solar cell	231
MoS_2_	2H → 1T	Li intercalation	Supercapacitor	238
WS_2_/Gr	2H → 1T	Li intercalation	Supercapacitor	239
MoS_2_	2H → 1T′	Li intercalation	Neuromorphic computing	241
WS_2_	2H →1T	Li intercalation	HER	243
ReS_2*x*_Se_2(1−*x*)_	2H →1T′	Li intercalation	HER	248
MoSe_2_	2H →1T	Li intercalation	HER	249

#### Application

The phase-transition of TMDs can be used in various applications, including wireless energy harvesting, solar cells, Li-ion batteries, resistive memory devices, and electrocatalytic applications.

**Fig. 13:**
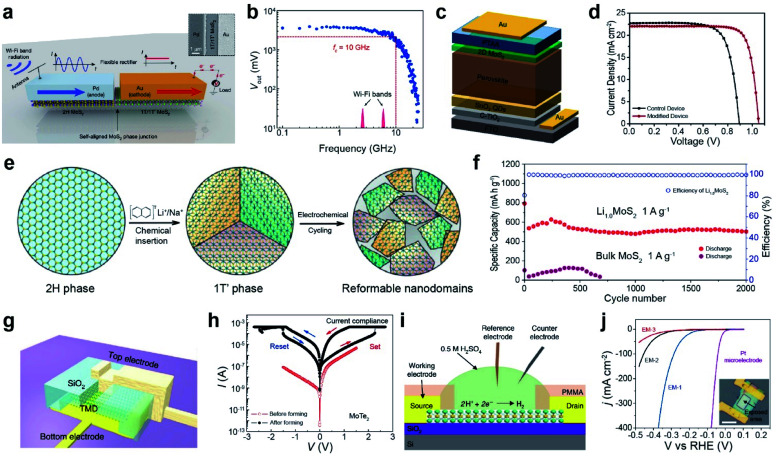
Phase transition for various applications: wireless energy harvesting, solar cell, Li-ion battery, resistive memory, and electrocatalytic applications. **Wireless energy harvesting:** (a) schematic of a lateral MoS_2_ semiconducting–metallic (2H–1T/1T′) Schottky diode with palladium and gold layers forming a Schottky and an ohmic contact, respectively. The antenna receives a Wi-Fi signal and converts it into an AC signal. The Schottky MoS_2_ diode rectifies the AC signal to the DC one. (b) Device output voltage as a function of frequency. **Solar cell:** (c) scheme of a solar cell architecture. (d) *J*–*V* curves of CsFAMA devices. **Li-ion battery:** (e) schematic of crystalline domain formation after chemical lithiation of bulk 2H-MoS_2_. (f) The cycling performance of Li_1.0_MoS_2_ and bulk MoS_2_ at 1 A g^−1^ for 2000 cycles, respectively. **Resistive memory:** (g) schematic of a vertical MoTe_2_ resistive random access memory (RRAM) device. (h) *I*–*V* curves of a MoTe_2_ RRAM device with a flake thickness of 24 nm and an area of (520 nm × 330 nm). Bipolar resistive switching behavior of MoTe_2_ before (red) and after (black) formation. Arrows indicate the sweep direction. **HER electrocatalyst:** (i) schematic of the electrochemical microcell for HER measurements. (j) Polarization curves of the current density obtained with EM-1, EM-2 and EM-3. (a and b) Adapted with permission.^229^ Copyright 2019, Springer Nature Limited. (c and d) Adapted with permission.^230^ Copyright 2020, Elsevier B.V. (e and f) Adapted with permission under a Creative Commons CC-BY License.^233^ Copyright 2016, American Chemical Society. (g and h) Adapted with permission.^240^ Copyright 2019, Springer Nature Limited. (i and j) Adapted with permission.^245^ Copyright 2018, Springer Nature Limited.

For wireless energy harvesting from the Wi-Fi band (2.45 and 5.9 GHz), a rectifier device is fabricated using MoS_2_ on a flexible Kapton substrate. Fig. 13a illustrates a lateral MoS_2_ semiconducting–metallic (2H–1T/1T′) Schottky diode with palladium and gold layers forming Schottky and ohmic contacts, respectively. Nonlinear *I*–*V* characteristics were observed for a given input radio frequency (RF) power owing to the presence of a Schottky junction between Pd and 2H-MoS_2_, which is attributed to the rectification behavior. For an input RF power of 5 mW, the device exhibited a cutoff frequency of 10 GHz and an output voltage of 3.5 V, because of the rectified voltage increase (*V*_out_) (Fig. 13b). Hence, a high cut-off frequency is sufficient to cover both Wi-Fi bands.^229^ A flexible Wi-Fi band antenna integrated with a MoS_2_ phase junction (2H–1T/1T′) Schottky diode can achieve wireless energy harvesting of electromagnetic radiation, which is efficiently used for self-powered systems.

A perovskite solar cell was fabricated with 1T-MoS_2_ as the hole transport layer (HTL) in the device architecture of FTO/c-TiO_2_/SnO_2_QD/Cs_0.1_FAPbI_3(0.81)_MAPbBr_3(0.09)_/MoS_2_/PTAA/Au (Fig. 13c). 1T-MoS_2_ reduced the mismatch between the energy-band alignment and trap density of perovskite, which increased the carrier concentration and improved the fill factor. The power conversion efficiency (PCE) of 1T-MoS_2_ increased from 15.05% to 18.54% (Fig. 13d).^230^ In other work, a single-layer MoS_2*x*_Se_2(1−*x*)_ nanosheet with the 1T phase (≈66%) was synthesized by electrochemical Li intercalation and exfoliation. The metallic 1T MoS_2*x*_Se_2(1−*x*)_ phase facilitates electron transport to the counter electrode surface in dye-sensitized solar cells (DSSCs).^231^ A higher PCE of 8.94% with 1T-MoS_2_@HCS (hollow carbon sphere) was attained compared to those of 2H-MoS2@HCS (8.16%) and Pt (8.87%).^232^

Metallic-phase TMDs are promising electrodes for metal-ion batteries. Electrochemical cycling for the phase transition from bulk 2H-MoS_2_ to 1T′-Li_*x*_MoS_2_*via* Li intercalation/deintercalation induced the formation of reformable nanodomains (Fig. 13e). Li_1.0_MoS_2_ showed a high specific capacity of 636 mA h g^−1^ at 1 A g^−1^ with a capacity retention of 80% and coulombic efficiency of 100%, whereas the capacity of bulk MoS_2_ collapsed very sharply (Fig. 13f). The domain boundaries between nanodomains facilitate the mass transport of Li^+^ ions and charge/discharge reaction: S + 2Li^+^ + 2e^−^ ↔ Li_2_S.^233^ Another example is the TiO–1T-MoS_2_ nanoflower composite for Na-ion batteries, which exhibits a high reversible capacity, attributed to the well-distributed conductive TiO with 1T-MoS_2_. This improves the electrical conductivity and stability.^234,235^ In Li–S batteries, metallic 1T-MoS_2_ nanodots suppressed the diffusion of polysulfides and accelerated redox kinetics, which was confirmed by *in situ* XRD and EIS characterization.^236^ In supercapacitors, metallic 1T-MoS_2_ displays an intrinsic capacitance of 14.9 μF cm^−2^, which is 10-fold higher than that of 2H-MoS_2_ in an aqueous electrolyte (1 M NaF).^237^ Metallic 1T-MoS_2_ nanosheets contributed to the efficient absorption/desorption of various aqueous electrolyte ions (H^+^, Na^+^, K^+^, and Li^+^), which resulted in a high capacitance (∼400 to 700 F cm^−3^).^238^ 1T-WS_2_/GO shows high performance in supercapacitors owing to the fast reversible reaction of W with proton insertion.^239^

A resistive random access memory (RRAM) device was fabricated by sandwiching TMD materials between top (Ti/Ni) and bottom (Ti/Au) electrodes on a SiO_2_ isolation layer, which ensured vertical transport (Fig. 13g).^240^ The switching behavior of MoTe_2_ was attributed to the formation of a conductive filament by the gradual phase transition from a 2H phase to a distorted transient (2H_d_; intermediate state) and T_d_ conductive orthorhombic (1T) phase with an applied electrical field. The *I*–*V* curves of a MoTe_2_ device (∼24 nm thick) demonstrate a resistive switching behavior after forming the conductive filament over 2.3 V (Fig. 13h). The device exhibited highly reproducible resistive switching within 10 ns between a low and a high resistive state. The thickness of MoTe_2_ varied with the set voltage of the device. In the case of the Mo_1−*x*_W_*x*_Te_2_ alloy, the set voltage can be reduced by increasing the concentration of W, as a result of the reduction in the energy barrier for the phase transition. The Au/Li_*x*_MoS_2_/Au device shows typical memristive behavior due to the reversible phase transition between 2H and 1T′-MoS_2_ films, which is controlled by the redistribution of Li^+^ ions under an electric field.^241^

Phase transitions are also useful for electrocatalytic applications. The basal planes of 2H-MoS_2_ and 2H-WS_2_ are effectively activated for the HER *via* a phase transition to the 1T phase.^242–244^ An electrochemical microcell was used to study the HER mechanism (Fig. 13i).^245^ The HER activity of 1T′ (EM-1) and a mixture of 1T′-2H (EM-2) and 2H (EM-3) phases was tested in an acidic medium. The LSV curves of EM-1 exhibited the highest HER activity, with a low onset potential of 200 mV and a high current density of 607 mA cm^−2^ at 400 mV compared to other samples (Fig. 13j). Another study demonstrated that the grain boundary between 2H and 1T phases acted as an active site for the HER.^246^ Furthermore, mesoporous 1T-MoS_2_ nanosheets with many edge sites and S vacancies provided superior HER activity owing to their high conductivity and abundant catalytically active sites.^247^ 1T-phase ReS_2*x*_Se_2(1−*x*)_ nanodots outperformed ReS_2_ and ReSe_2_ in terms of the HER performance.^248^ Superior HER performance was also observed for molybdenum dichalcogenides (MoSe_2_) over their tungsten counterparts (WS_2_ and WSe_2_) after BuLi exfoliation.^249^ In photocatalytic H_2_ evolution, the lateral 1T@2H-MoS_2_ heterostructure demonstrates an improved photocatalytic efficiency because the 1T phase serves as an electron acceptor and transporter to suppress a charge recombination process.^225^ During the HER, the 1T phase in the hybrid TiO_2_@1T-MoS_2_ is irreversibly converted into a more active 1T′ phase, providing more active sites and improving the HER activities.^250^

### 3.2. Heterostructures

Heterostructures are essential elements in the modern semiconductor industry and play a crucial role in high-speed electronics and optoelectronic devices.^251–256^ This wide range of applications stem from the tunable band alignment which enables electron and hole transfer across the heterojunction. TMD-based heterostructures consist of vertical van der Waals and lateral heterostructures.^257–261^ The former is constructed by the layer-by-layer stacking of 2D TMD materials, and the latter is formed by the lateral growth of another TMD at the edge of an initial TMD. The construction of a vertical heterostructure by applying the “pick-up” and “drop-down” methods with the mechanical exfoliation approach has been described in another study.^262–267^ The direct growth of heterostructures on pristine TMDs by employing CVD is focused on in this review.

#### Vertical heterostructures

**Fig. 14:**
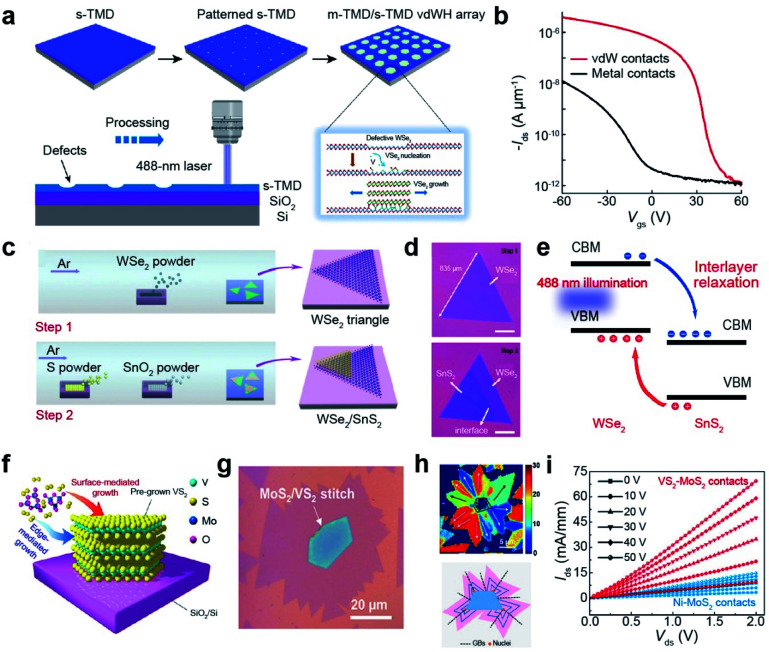
Defect and edge mediated growth for vertical van der Waals heterostructures (vdWH) and lateral heterostructures. **Vertical van der Waals heterostructures:** (a) schematic demonstration of large-area WSe_2_ selectively patterned to create periodic defect arrays as nucleation sites for site-specific growth of VSe_2_ to form VSe_2_/WSe_2_ vdWH arrays. (b) *I*–*V* transfer curves of VSe_2_/WSe_2_ vdWH compared with a WSe_2_ FET, obtained at *V*_DS_ = −0.1 V. (c) Schematics of a two-step epitaxy growth of the WSe_2_/SnS_2_ heterostructure. (d) Corresponding optical image of as-grown monolayer WSe_2_ obtained after step 1 and the as-grown WSe_2_/SnS_2_ vertical heterostructure after the second step by using the same WSe_2_ flake. (e) Schematic illustrations of the band structure and photoexcitation processes at the type-III WSe_2_/SnS_2_ heterojunction. **Lateral heterostructures:** (f) schematics of MoS_2_ growth at the edge or surface of the pre-synthesized VS_2_ nanosheets. (g) Optical images of the VS_2_/MoS_2_ stitching. (h) SHG imaging showing the distribution of MoS_2_ domains surrounding VS_2_ nanosheets (top) and proposed illustration of the stitching growth mechanism of MoS_2_ at the vertices of VS_2_ flakes (bottom) (blue lines indicate the growth pattern of MoS_2_). (i) *I*_DS_–*V*_DS_ characteristics of the VS_2_/MoS_2_ device compared with the Ni-MoS_2_ contact, at varying *V*_G_ (0 to 50 V). (a and b) Reproduced with permission.^268^ Copyright 2020, Springer Nature Limited. (c–e) Reproduced with permission under a Creative Commons CC-BY License.^270^ Copyright 2017, Springer Nature Limited. (f–i) Reproduced with permission.^271^ Copyright 2018, American Chemical Society.

To grow vertical heterostructures directly, a nucleation site is required to initiate an overlayer growth on top of a pristine TMD. With the aid of focused laser irradiation combined with raster scanning, a periodic array of defects on pristine WSe_2_ is created to serve as nucleation sites (Fig. 14a).^268,269^ Direct laser patterning enables the creation of local defects at specific sites without contaminating other areas of the underlying WSe_2_. The pre-patterned WSe_2_ was placed in a separate furnace to synthesize an overlayer of metallic VSe_2_. The growth temperature of VSe_2_ was 600 °C (much lower than that of WSe_2_ at 850 °C), to prevent thermal damage to WSe_2_. The metallic VSe_2_/WSe_2_ heterostructure significantly reduced the contact resistance between electrodes and WSe_2_ by forming an atomically clean vdW interface, resulting in a high on/off ratio of 10^7^ and a high “on” current (Fig. 14b). These studies demonstrated that synthetic VSe_2_/WSe_2_ vdW contacts offer considerable advantages over typical lithographically developed electrodes with low contact resistance.^268^

Furthermore, unstable edges or vertices of the TMD flakes can be utilized as nucleation seed sites. As an example, epitaxial growth of the SnS_2_ overlayer from a vertex of a triangular WSe_2_*via* a two-step CVD process flake was observed in the optical image, forming vdW WSe_2_/SnS_2_ vertical bilayer heterostructures (Fig. 14c and d).^270^ A type-III heterojunction between WSe_2_ and SnS_2_ was established (Fig. 14e). Therefore, photo-excited electrons and holes in WSe_2_ prefer to transfer to low-energy states in SnS_2_, rather than forming excitons in WSe_2_, resulting in significant PL quenching of WSe_2_.

#### Lateral heterostructures

Two-step lateral heterostructure growth of pre-synthesized VS_2_ nanosheets, followed by stitching with MoS_2_ monolayers at its edges, is shown in Fig. 14f and g.^271^ To uncover the nature of the proposed epitaxial growth behavior of lateral VS_2_/MoS_2_ stitching, second harmonic generation (SHG) imaging was employed. The orientation distribution of the surrounding MoS_2_ domains reveals the polycrystalline nature of MoS_2_ domains (Fig. 14h (top)). Nucleation arises from the vertices of VS_2_, instead of lateral epitaxial growth from the VS_2_ edges (Fig. 14h (bottom)). In FET devices with lateral heterostructures, *I*_DS_ with VS_2_ contact is six times higher than that of the counterpart with the Ni contact (for the same *V*_G_ and *V*_DS_), implying that this approach is a promising way to reduce contact resistance (Fig. 14i).

Overall, this section consolidates recent advances in post-processing techniques for the synthesis of lateral and vertical heterostructures. By considering the respective thermal decomposition temperatures of TMDs, a vast number of vdW heterostructures can be synthesized. A summary of the growth of these heterostructures is given in Table 3. vdW heterostructures grown in a one-step process using mixed precursors have been reported; however, there is a high probability of forming alloys or mixed heterostructures with this method, degrading the unique physical and chemical properties of heterostructures.

**Table 3:** Summary of the growth heterostructures by applying CVD

Host	Incorporated material	Processing temperature	Heterostructure type	Reference
WS_2_	NbS_2_	750–800 °C	Vertical and lateral	251
WS_2_	CuI nanosheets	450 °C	Vertical	252
WS_2_	Sb_2_Se_3_ nanowires	600 °C	Vertical	253
WS_2_	FAPBI_3_ (2D perovskite)	360 °C	Vertical	254
WSe_2_	CoSe	535 °C	Lateral	255
WS_2_	CdI_2_	315–325 °C	Vertical	256
WS_2(1−*x*)_Se_2*x*_	SnS_2_	550 °C	Vertical	257
MoS_2_	Sb_2_Te_3_	600 °C	Vertical	258
MoS_2_	MoSe_2_	600 °C	Vertical	259
MoS_2_	NiTe_2_	550 °C	Vertical	260
MoSe_2_	InSe_2_	660 °C	Vertical	261
MoSe_2_	GaSe	710 °C	Vertical and lateral	273

#### Application

Heterostructures are desirable in a broad range of fields, including field-effect transistors, biosensors, light-emitting diodes, photodetectors, photovoltaic devices, and energy storage.

**Fig. 15:**
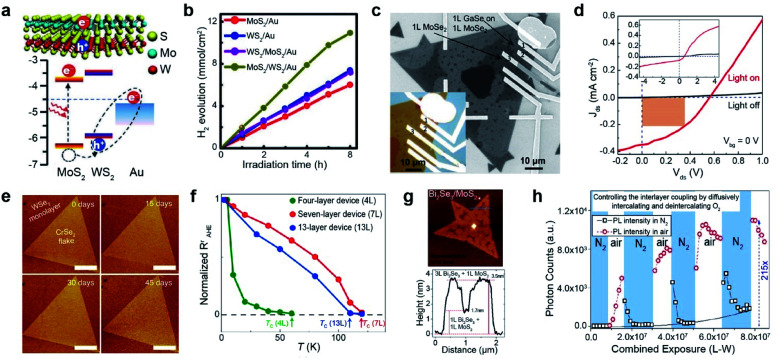
Applications of heterostructures: photocatalysis, solar cell, magnetism, and sensors. **Photocatalysis:** (a) effective electron transfer mechanism for enhanced photocatalytic H_2_ evolution in MoS_2_/WS_2_ vertical heterostructures under light irradiation. (b) Photocatalytic H_2_ evolution curves of MoS_2_, WS_2_, MoS_2_/WS_2_, and WS_2_/MoS_2_ under an irradiation time of 6 h. **Solar cell:** (c) SEM image of a device consisting of a 1 L GaSe/MoSe_2_ heterostructure. Marked positions 1 and 2 indicate GaSe/MoSe_2_, while position 3 is for MoSe_2_. Inset shows the corresponding optical image. (d) *J*_DS_–*V*_DS_ curves (*V*_bg_ = 0 V) with (red solid curve) and without (black solid curve) white light illumination across the heterojunction. The area with orange shading indicates *P*_max_. The inset shows the *I*_DS_–*V*_DS_ curves on a larger scale. **Magnetism:** (e) atomic force microscopy images of monolayer CrSe_2_ on WS_2_ after exposure in air up to 45 days (scale bars: 3 μm). (f) Remnant anomalous Hall resistance (*R*^r^_AHE_) as a function of temperature with 4, 7, and 13 layers of CrSe_2_. **Sensors:** (g) AFM image of the Bi_2_Se_3_/MoS_2_ 2D heterostructure and corresponding line profile, demonstrating the growth of Bi_2_Se_3_ on MoS_2_ crystals. (h) Variation of PL intensity under alternating air and nitrogen environments while a focused laser is applied, demonstrating that a nitrogen environment restores the interlayer coupling. (a and b) Reproduced with permission.^272^ Copyright 2016, Wiley-VCH. (c and d) Reproduced with permission.^273^ Copyright 2016, American Association for Advancement of Science. (e and f) Reproduced with permission.^274^ Copyright 2021, Springer Nature Limited. (g and h) Reproduced with permission.^275^ Copyright 2019, American Chemical Society.

MoS_2_/WS_2_ and WS_2_/MoS_2_ vertical heterostructures (type-II heterojunctions) were explored as photocatalysts for hydrogen evolution (Fig. 15a).^272^ For MoS_2_/WS_2_/Au, the photoexcited electrons in WS_2_ were injected into the conduction band of MoS_2_*via* stepwise band alignment and contributed to the reduction of H^+^ to evolve H_2_, whereas the holes in the monolayer WS_2_ were neutralized by electrons from the electrode. Such effective separation of the photo-excited electron–hole pairs in the MoS_2_/WS_2_ stacks greatly promoted H_2_ evolution, leading to the highest H_2_ evolution content after 6 h (Fig. 15b).

A vertically stacked vdW GaSe/MoSe_2_ heterostructure was constructed to create a p–n junction for photodetector and solar-cell applications (Fig. 15c).^273^ To test the photovoltaic response of vdW GaSe/MoSe_2_, a FET was fabricated. The *I*–*V* transfer characteristics reveal diode behavior, which is ascribed to p-type GaSe and n-type MoSe_2_. Furthermore, the photovoltaic characteristics of the heterostructure under white light illumination were investigated. While negligible photoresponse (black solid curve) is observed in the dark, the output current (red solid curve) under light shows an open-circuit voltage (*V*_oc_) of ∼0.57 V and a short circuit current density (*J*_sc_) of ∼0.35 mA cm^−2^ at *V*_bg_ = 0 V (Fig. 15d). Ultimately, the resulting solar energy conversion performances, such as photo-to-electron conversion efficiency, fill factor, and photoresponsivity, are estimated to be 0.12%, 0.38 and 5.5 mA W^−1^, respectively, at *V*_bg_ = 0 V.

The poor stability of TMDs under ambient conditions is often a bottleneck for scalable device applications. Recent studies have shown that 2D TMD heterostructures can significantly improve their air stability through interlayer coupling. 2D CrSe_2_ nanosheets, with varying thicknesses (down to a monolayer), were grown on a dangling bond-free WSe_2_ monolayer *via* a two-step CVD process.^274^ Atomic force microscopy images of the heterostructures revealed that monolayer CrSe_2_ exhibited an outstanding air stability for up to 45 days (Fig. 15e), with no apparent change in the surface roughness or magnetic properties. Theoretical calculations suggested that charge transfer for the WSe_2_ substrate and interlayer coupling within CrSe_2_ play a critical role in the magnetic order of few-layered CrSe_2_ nanosheets. Notably, magnetotransport measurements revealed that the layer thickness of CrSe_2_ exhibits differential magnetic properties. A thickness of up to three layers exhibits weak magnetic characteristics, whereas an increase in ferromagnetic (FM) properties was observed for four layers.^274^Fig. 15f shows that the remnant anomalous Hall resistance (*R*^r^_AHE_) of the layer thickness of CrSe_2_ decreases with increasing temperature and vanishes at around the Curie temperature (*T*_C_). The *T*_C_ values of the 7 and 13 L devices were found to be significantly higher than those of the 4 L device, indicating that the FM properties increased significantly with layer thickness.

Finally, the interlayer coupling of post-deposited Bi_2_Se_3_ on MoS_2_ heterostructures can be modulated by regulating the presence of oxygen with controlled thermal energy.^275^Fig. 15g shows an AFM image of a Bi_2_Se_3_/MoS_2_ heterostructure with a height of 3.5 nm. While the PL intensity is significantly quenched under a N_2_ atmosphere, it increases in air (Fig. 15h), indicating that intercalated oxygen interrupts the interlayer coupling between Bi_2_Se_3_ and MoS_2_. This characteristic can be applied to gas sensors.

## 4. Summary and prospects

We reviewed the recent developments and state-of-the-art atomic and structural modifications of TMDs with their unique physical/chemical properties. High performance device applications were discussed for post-treated TMDs, such as electronics, catalysis, energy storage, wearable biosensors, piezoelectricity, CoVID-19 sensors, TENGs, flexible PNGs, superconductor devices, wireless energy harvesting, solar cells, and memory and neuromorphic devices. The outlook for each topic is as follows.

### 4.1. Selectivity and scalability of vacancy generation

Expansion from a laboratory to an industrial scale is critical. Therefore, homogeneous vacancy generation by using a cost-effective process over a large area of TMDs should be considered. During post-treatment of TMDs, unintended defects such as the coexistence of metal and chalcogen vacancies, line defects and degradation can be generated under high power or temperature. Selective vacancy engineering plays a critical role in the research of the effect of chalcogen vacancies on the properties of TMDs. More research is needed to improve selective vacancy generation (S_V_ or TM_V_).

### 4.2. Unclear catalytic mechanism by spontaneous oxygen passivation at vacancy sites

Several studies have been conducted to enhance the electrocatalytic performance owing to chalcogen vacancies in TMDs. However, STEM analysis of chalcogen vacancies has revealed that oxygen passivation at the vacancies is unavoidable due to spontaneous incorporation of oxygen. The actual active sites for catalytic reactions are still ambiguous. DFT calculations have been successfully used to analyze the Gibbs free energy of the HER and adsorption of metal ions on the vacancies. Most structural models are monolayers, whereas synthesized energy conversion and storage materials are multilayers. Therefore, a comparison of experimental results with theoretical ones shows apparent differences. A precise model that is consistent with multilayer materials is required. Moreover, *in situ*/*operando* characterization and advanced techniques are needed to understand the intermediate reactions and electrochemical reactions on vacancies for more diverse applications.

### 4.3. Dopant's homogeneity and its position

Studies on achieving substitutional impurity doping in TMDs have progressed considerably over the past year. Several innovative techniques for incorporating dopants have been studied; however, a few challenges remain. Efforts to modulate and control the homogenous distribution of dopants are still daunting. As the homogeneity of dopants varies from the basal planes to the edges, the physical and chemical properties of the doped TMD tend not to be uniform. Therefore, a critical look is required during the generation of defects to ensure the uniformity of the defect distribution, as they will serve as nucleation sites for dopants. Furthermore, the control of the dopant position is very important for some applications, such as single-photon emission and diluted magnetic 2D semiconductors, but it has not yet been resolved.

### 4.4. Broader research for Janus 2D materials

The progress is still in its infancy. Although several theoretical predictions have been performed for numerous Janus structures, only MoSSe and WSSe have been experimentally investigated. Furthermore, the uniqueness of the Janus structures should trigger new breakthrough applications in the field of science. Therefore, efforts should be made to improve the fabrication skills and applications of Janus 2D TMDs in the future.

### 4.5. Hybrid and asymmetric functionalization

In covalent functionalization approaches, the high coverage and uniform distribution of functional molecules on TMDs are limited, and hence, they deserve special attention. A special focus on the functionalization of Janus or heterostructure TMDs with organic molecules is needed, which will eventually offer some interesting physical and chemical properties based on the functional molecules. In addition, hybrid and asymmetric/Janus functionalization of TMDs have recently provided enriched electronic and optical properties of pristine TMDs.^276^ Therefore, this will facilitate the fabrication of next-generation multi-storage memory devices.

### 4.6. Optimization of functionalization techniques

Conventional techniques (drop-casting, dip-coating or soaking, spin-coating, and thermal evaporation) are predominantly used for organic functionalization of TMDs.^277–280^ These techniques consist of some disadvantages such as thick coatings, non-uniformity, aggregation, solvent evaporation dynamics, uncontrollable surface dewetting, and material accumulation at drop edges (coffee ring effect). Spin-coating has many adjustable parameters (concentration of organic molecules, boiling point of the solvent, and rotation speed) to optimize.^281–283^ In the future, direct printing can be applied to overcome many issues found in other techniques. Currently, functional organic molecules are dispersed in solvents to form the desired functional ink. As a result, the integration of organic molecule printing techniques with TMDs can be effectively used for large-scale fabrication at low-cost, lightweight wearable biosensors, flexible photodetectors, micro-energy storage devices, and memory elements.^284–287^ Therefore, it will provide a pathway for developing next-generation electronic technologies.

### 4.7. Unclear repair mechanism

Currently, few methods are employed to repair atomic chalcogen vacancies in TMD materials. The repair mechanism for healing TMDs with organic molecules (*i.e.* thiol molecules and bis(trifluoromethane) sulfonamide (TFSI)) are still unclear.^187^ Therefore, more research, using advanced techniques such as STEM and *in situ* TEM, is required.

### 4.8. Quantum application for oxygen passivation

Furthermore, passivating TMDs with oxygen atoms showed promising applications in electronic devices. Similarly, oxygen passivation offers some interesting quantum phenomena such as elevated electron–phonon interaction, breaking structural symmetry and anisotropy, Rashba spin–orbit interaction, enhanced piezoelectricity, and improved Ising superconductivity.^198^

### 4.9. Stability and scalability of phase transition

Phase engineering has been studied to prepare metallic (1T or 1T′) phases from semiconducting (2H) phases. In the field of energy applications, the most widely studied technique for phase transition is Li intercalation through Li or organolithium reagents, which are highly corrosive and flammable, hindering industrial scalability. Furthermore, long lithiation times at high temperatures result in excess lithium and organic residues. The thermodynamic instability of the synthesized 1T/1T′ phase is a major drawback that hinders its further application. Thus, the yield and stability of the 1T/1T′ phase are important challenges that need to be overcome. Finally, it is necessary to develop a different phase-selective route to prepare metallic TMDs with a high phase purity.

### 4.10. Large scale synthesis of vertical heterostructures

The scalable growth of heterostructures has been a challenge, restricting extensive applications. At present, high-quality vdW heterostructures with sizes of only a few micrometers are achievable. The fabrication of large-area 2D heterostructures has been reported by sequential atomic layer deposition, molecular beam epitaxy, and exfoliation transfer techniques. However, clean and sharp interfaces were not obtained using these fabrication methods. Hence, continuous research on the realization of high-quality vdW heterostructures is necessary for the development of novel optoelectronic, electronic, and solar-cell devices. CVD is one of the most promising methods for producing high-quality, large-size TMDs. This is because of the ease in designing the growth parameters and revamping home-built CVD systems.^288^ In the future, further advances are required to improve the synthesis of wafer-scale high-quality vdW heterostructures for manufacturing lines in the semiconducting industry.

## Author contributions

B. K., L. A. A., S. M. K. and K. K. K. designed the scope of the review. B. K., Y. S. W., L. A. A. and S. H. C. collected the references, organized the images and wrote the initial manuscript. Y. S. W. and S. H. C. modified and arranged the figures. S. M. K. and K. K. K. supervised and revised the manuscript. All the authors participated in the revision of the manuscript.

## Conflicts of interest

There are no conflicts to declare.

## Supplementary Material
